# A complex mode of aggressive mimicry in a scale-eating cichlid fish

**DOI:** 10.1098/rsbl.2015.0521

**Published:** 2015-09

**Authors:** Nicolas Boileau, Fabio Cortesi, Bernd Egger, Moritz Muschick, Adrian Indermaur, Anya Theis, Heinz H. Büscher, Walter Salzburger

**Affiliations:** Zoological Institute, University of Basel, Vesalgasse 1, Basel 4051, Switzerland

**Keywords:** aggressive mimicry, lepidophagy, molecular diet analysis, colour vision, *Plecodus straeleni*, Lake Tanganyika

## Abstract

Aggressive mimicry is an adaptive tactic of parasitic or predatory species that closely resemble inoffensive models in order to increase fitness via predatory gains. Although similarity of distantly related species is often intuitively implicated with mimicry, the exact mechanisms and evolutionary causes remain elusive in many cases. Here, we report a complex aggressive mimicry strategy in *Plecodus straeleni*, a scale-eating cichlid fish from Lake Tanganyika, which imitates two other cichlid species. Employing targeted sequencing on ingested scales, we show that *P. straeleni* does not preferentially parasitize its models but—contrary to prevailing assumptions—targets a variety of co-occurring dissimilar looking fish species*.* Combined with tests for visual resemblance and visual modelling from a prey perspective, our results suggest that complex interactions among different cichlid species are involved in this mimicry system.

## 1. Introduction

Following its original discovery in tropical butterflies, numerous instances of mimicry have been described, constituting textbook examples of adaptation by natural selection and striking cases of evolutionary convergence [1,2]. The various mimicry strategies can broadly be classified, according to function, into ‘protective’, ‘reproductive’ and ‘aggressive’ [2,3]. In the latter case, a predatory or parasitic species resembles a harmless model in order to dupe potential victims, thereby increasing the chance of successful attacks. As for all mimicry systems, this only works under a set of conditions: (i) mimics must be rare relative to their models, (ii) they must gain an advantage when associated with their models, (iii) the mimicked traits should closely match the model's and (iv) their geographical ranges and habitats should overlap [4,5]. Aggressive mimicry seems to be especially prevalent in fishes [6], which can employ three different tactics to deceive their victims: (i) mimics imitate small ‘beneficial’ models in order to parasitize larger prey [7–9], (ii) mimics school with harmless models to launch attacks against smaller prey [10] and (iii) mimics become ‘wolves in sheep's clothing’ to exclusively attack similarly sized models, as observed, for example, in some fin-biting and scale-eating fish species [6], but not in others, like the bluestriped fangblenny, *Plagiotremus rhinorhynchos* [9].

Feeding on the scales of other species (‘lepidophagy’) ranks among the most curious foraging strategies in fishes, known from a few families only [11,12]. It is common, however, in the exceptionally diverse adaptive radiations of cichlid fishes in East Africa. Lake Tanganyika, for example, is home to six lepidophagous cichlid species [13], which show remarkable adaptations such as hook-like teeth and asymmetry (‘handedness’) of mouth opening [14].

**Figure 1.**
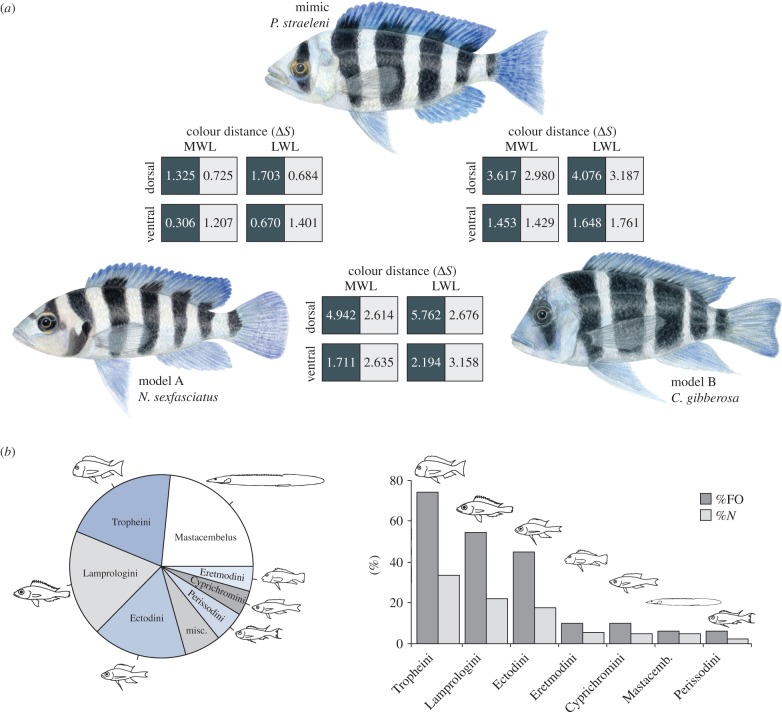
(*a*) The mimic *P. straeleni*; the models *N. sexfasciatus* (model A) and *C. gibberosa* (model B) (drawings by Lucretia Ehrensperger). Colour distances Δ*S* between each pair and the four body regions (dark bars, light bars, dorsal and ventral) from the perspective of typical Tanganyikan-cichlid long and middle wavelength visual systems [16] are denoted. Dark and light squares show Δ*S* for dark and light bars between species. MWL: middle wavelength; LWL: long wavelength. Note that mimic–model pairs appear more similar (smaller values) to one another than the model–model pair. (*b*) Prey spectrum of *P. straeleni* in relative frequency across all scales identified in stomachs (diagram to the left). %FO: frequency of occurrence of prey; %*N*: average per cent number (average representation of a given prey within one stomach). Outlined fishes are the most representative species within each tribe.

The specific coloration patterns of some Tanganyikan scale-eaters have been implicated with aggressive mimicry [6,13,15]. *Plecodus straeleni*, for example, was suggested to mimic and prey upon several co-occurring models [15]. In southern Lake Tanganyika, *P. straeleni* features a characteristic bluestriped pattern (figure 1*a*) and bears a close resemblance in size, body shape and coloration to two other syntopic cichlid species: *Neolamprologus sexfasciatus* and *Cyphotilapia gibberosa* (figure 1; electronic supplementary material, Movie S1). It remains unclear, however, whether *P. straeleni* forages predominantly on its models, as was hypothesized in [6], or whether it preys upon a wider range of non-resembling species.

We combined molecular diet analysis and morphological assessments to investigate the feeding behaviour of *P. straeleni* and to evaluate its resemblance to the two putative model species. We first used a PCR-based method to assign single ingested scales to specific prey species. Since scales are discrete units, barcoding allowed us not only to identify prey items but also to quantify the relative prevalence of different prey species in the diet of the mimic. Using this unique approach, we overcame the main limitations of previous studies on scale-eating fish that relied on behavioural observations or morphological classifications of ingested scales alone [6,17]. We then used theoretical fish visual models and a body colour score from a large set of Tanganyikan cichlids to assess how well the mimic would resemble its models compared to other Tanganyikan fish species. Finally, we quantified the relative abundance of the mimic and its models using transect counts.

## 2. Material and methods

Specimens of the target species were collected on SCUBA in the southern part of Lake Tanganyika (electronic supplementary material, table S1). The intestinal tracts of 38 *P. straeleni* individuals were dissected in the field and preserved in ethanol. In the laboratory, stomach and gut contents were removed and the DNA of individual ingested scales was extracted. We developed a PCR-based approach to assign single ingested scales to specific prey species by sequencing fragments of the mitochondrial ND2 gene, effectively repressing the amplification of endogenous DNA with *P. straeleni*-specific blocking oligonucleotides. For non-cichlid scales found in two stomachs, we sequenced a part of the mitochondrial COI gene. Sequences were inspected by eye and aligned to a reference ND2 dataset from 180 cichlid species representing approximately 90% of all Tanganyikan cichlid species. To assign individual scales to species, we used first blast (http://blast.ncbi.nlm.nih.gov/) and then phylogenetic analyses for species that could not be unambiguously identified with blast (electronic supplementary material, table S2). To quantify diet composition, we used the indexes of frequency of occurrence (%FO) and average per cent abundance (%*N*) [18] (figure 1*b*).

Spectral measurements of different body regions of *P. straeleni* (*N* = 8; mimic), *N. sexfasciatus* (*N* = 2; model A) and *C. gibberosa* (*N* = 1; model B) (electronic supplementary material, figure S1) were combined with theoretical visual models [19,20] to assess the similarity between mimic and models in terms of colour distance (Δ*S*). Δ*S* was modelled using mid-wavelength-shifted (green) and long-wavelength-shifted (red) visual templates, representing the two prevalent visual systems in Tanganyikan cichlids [16]. Thereby, Δ*S* = 1 is an approximate threshold of discrimination, Δ*S* < 1 indicates colours are chromatically indistinguishable, and ΔS > 1 indicates colours are discriminable from one another [19,20]. A comparative body colour scoring of the mimic and models was also used to determine the resemblance to an additional 68 co-occurring Tanganyikan cichlid species (as per [21]) (electronic supplementary material, tables S3 and S4). Additionally, we performed a transect survey to estimate the relative abundance of the mimic and its models. At each site (*N* = 7), we counted the number of mimics and models in a linear transect running from the shoreline to a depth of 25 m (electronic supplementary material, table S5).

Data, statistical approaches and detailed methods are available from the electronic supplementary material.

## 3. Results

We processed a total of 815 scales from 38 *P. straeleni* intestinal tracts, of which 469 could be successfully amplified, sequenced and taxonomically assigned (electronic supplementary material, table S2). Phylogenetic analyses resulted in the identification of 43 prey species from virtually all Tanganyikan cichlid lineages (10 out of 12 ‘tribes’). Scales of up to nine different species were found within a single stomach and diet overlap between individuals ranged from 0.5 to 15% (or from 4 to 38% when grouped into tribes). Overall, the ingested scales reflected the species composition from the rocky habitat of Lake Tanganyika, with the majority of identified scales belonging to the three most abundant Tanganyikan cichlid lineages (Tropheini: 20% of all scales (*N* = 111); Lamprologini: 19%, *N* = 103; Ectodini: 17%, *N* = 94) plus the endemic spiny eel (*Mastacembelus ellipsifer*: 23%, *N* = 123, figure 1*b*). Importantly, only five scales turned out to belong to the model species *C. gibberosa* (*N* = 1, 0.2%) and *N. sexfasciatus* (*N* = 4, 0.7%).

In terms of visual resemblance, we found that, regardless of the visual system, *P. straeleni* closely resembled *N. sexfasciatus*, with Δ*S* values being either below or just around the discrimination threshold for all body regions (Δ*S*, 0.3–1.7, mean ± s.e. = 1.0 ± 0.2). Moreover, although the match for *P. straeleni* and *C. gibberosa* was not as striking (Δ*S*, 1.4–4.1, 2.5 ± 0.4), the mimic–model pair always appeared more similar from the cichlids eye's perspective than the model–model pair (Δ*S*, 1.7–5.8, 3.2 ± 0.5, figure 1*a*). This agrees with the comparative analysis of body coloration in Tanganyikan cichlids, which clustered together *P. straeleni* and its model species in the colour morphospace (electronic supplementary material, figure S2). The transect survey revealed one to five individuals of *P. straeleni* (mean 2.1 ± 1.5), 9–35 (19.4 ± 9.8) *N. sexfasciatus* and one to five (1.3 ± 2.2) *C. gibberosa* per site, resulting in an average ratio of models to mimics of 10.2 (±3.6) (electronic supplementary material, table S5).

## 4. Discussion

This study represents—to the best of our knowledge—the first molecular diet analysis in a lepidophagous fish. The sequencing of short fragments (imposed by poor DNA quality of ingested scales) proved to be adequate for barcoding at the species level [22]. Rates of contamination by exogenous DNA and sequencing failure were minor compared with the precision in quantifying and identifying prey composition. A significant detection bias seems unlikely, since we were able to identify 43 cichlid species plus the endemic spiny eel and up to nine distinct prey species in a single digestive tract*.* Comparing diet overlaps between individuals showed that there is a great variability between stomach contents in terms of prey species and number of scales per species. However, since stomach contents are snapshots of fish diets [23], we were unable to conclude if there is geographical prey specialization in *P. straeleni*.

Overall, we found that *P. straeleni* has a rather opportunistic feeding strategy, which contradicts the prevailing view that it primarily attacks its models [6,15]. Yet, these findings are in line with previous field observations suggesting a ‘camouflaging effect’ in *P. straeleni* [13,17].

The visual models and colour scoring (mimics should closely match models), in addition to transect data (mimics must be rare compared with models and their habitat should overlap), support the aggressive mimicry hypothesis. However, our findings do not support the prediction stated in [6] for fin-biting and scale-eating fish, in that *P. straeleni* does not preferably attack its similar sized models, but a broad spectrum of species of all sizes, as it is the case for *P. rhinorhynchos* [8,9]. Resembling common sympatric cichlid species may thus provide predatory advantages over a broad scale of prey species. However, to ascertain aggressive mimicry of *P. straeleni*, future studies should also consider other aspects of visual resemblance such as similarity in luminance and pattern of visual signal [24]. Additionally, behavioural experiments are needed to show an increased foraging success of *P. straeleni* when in proximity to its models (e.g. [10]). Though difficult to set up, such assays could answer the question whether resemblance to two distinct model species serves two different mimicry strategies: aggressive and protective (as has been shown in coral reef fish [3]). There are indeed reports that *P. straeleni* blend in schools of *Cyphotilapia frontosa* (the similarly coloured congener to *C. gibberosa* from the northern part of Lake Tanganyika) [15], suggesting a protective mimicry function.

While aggressive mimicry systems are usually composed of three species—the mimic, its model and a dupe (or receiver) [2]—mimicry in *P. straeleni* apparently involves two models and a whole species community as dupes, making it one of the most complex aggressive mimicry systems known to date.

## Supplementary Material

Supplemental Tables, Figures and Movie

## Supplementary Material

Boileau_et_al_media.docx

## Data accessibility

DNA sequence data of ingested scales have been deposited in Dryad: (http://dx.doi.org/10.5061/dryad.cm02v).
