# Supplementary material for: A complex mode of aggressive mimicry in a scale-eating cichlid fish
Source: Biol Lett. 2015 Sep;11(9):20150521. doi: 10.1098/rsbl.2015.0521 (PMC4614428; doi:10.1098/rsbl.2015.0521)
Supplement: Supplemental Tables, Figures and Movie [file rsbl20150521supp1.docx]

**Electronic Supplementary Material**

**A Complex Mode of Aggressive Mimicry in a Scale-eating Cichlid Fish**

Nicolas Boileau, Fabio Cortesi, Bernd Egger, Moritz Muschick, Adrian Indermaur, Anya Theis, Heinz H. Büscher and Walter Salzburger

**Supplemental Tables, Figures and Movie**

Table S1. Specimens used for stomach content analyses

Table S2. Colour traits definitions (joint as external file: Table_S2.txt)

Table S3. Colour traits values (joint as external file: Table_S3.txt)

Table S4. Transect survey counts

Table S5. Molecular diet analysis results

Table S6. GenBank Accession Numbers of Cichlid ND2 reference data set

Figure S1. Spectral reflectance curves

Figure S2. Colour scoring of Tanganyikan cichlids

Movie S1

**Supplemental Experimental Procedures**

Analysis of visual resemblance

Transect survey

Detailed molecular methods

Diet analysis

**Supplemental ReferencesSupplemental Tables and Movie**

**Table S1. List of specimens used for stomach content analyses**

*P. straeleni Ref. Nr.:* Reference number of *P. straeleni* stomach*.*

*Total scales:* total number of scales found in stomach.

*Processed:* number of scales that were analysed.

*Cichlid:* number of uncontaminated sequences belonging to cichlids.

*Contaminated*: numbers of sequences that are considered as contaminated with *P. straeleni* gut DNA.

*Masell:* Number of sequences belonging to the spiny eel *Mastacembelus ellipsifer*.

*Failed:* number of scales for which we could not retrieve a cichlid ND2 sequence.

*Date (YYYYMMDD):* Date of sample collection.

*Location:* name of the area where sample was collected including coordinates.

*SL:* standard length (mm).

*TL:* total length (mm).

*Weight* of *Plecodus* individual (g).

| *P. straeleni* Ref. Nr. | Total scales | Processed | Cichlid | Contaminated | *Masell* | Failed | Date | Location Name | Coordinates SOUTH | Coordinates EAST | SL | TL | Weight | Sex |
| --- | --- | --- | --- | --- | --- | --- | --- | --- | --- | --- | --- | --- | --- | --- |
| 21D6 | 24 | 24 | 19 | 0 |  | 5 | 20080915 | Mbita Island NW | 8.74988 | 31.08747 | 73 | 88 | 8.5 | n/a |
| 36A9 | 36 | 36 | 27 | 2 |  | 7 | 20100222 | Mbita Island W | 8.75323 | 31.08457 | 82 | 101 | 17 | f |
| 48B9 | 13 | 13 | 10 | 3 |  | 0 | 20100306 | Wonzye Point | 8.72519 | 31.13338 | 83 | 103 | 17.9 | f |
| 21D5 | 12 | 12 | 11 | 0 |  | 1 | 20080915 | Mbita Island NW | 8.74988 | 31.08747 | 72 | 90 | 10.5 | n/a |
| 36H2 | 43 | 43 | 35 | 2 |  | 6 | 20100223 | Kasakalawe Lodge | 8.78107 | 31.09151 | 86 | 118 | 18.1 | n/a |
| 36H4 | 36 | 36 | 20 | 9 |  | 7 | 20100223 | Kasakalawe Lodge | 8.78107 | 31.09151 | 73 | 92 | 11 | n/a |
| 36B1 | 8 | 8 | 1 | 4 |  | 3 | 20100222 | Mbita Island W | 8.75323 | 31.08457 | 72 | 89 | 10.8 | n/a |
| 36H3 | 2 | 2 | 1 | 1 |  | 0 | 20100223 | Kasakalawe Lodge | 8.78107 | 31.09151 | 67 | 82 | 9 | n/a |
| 36A7 | 19 | 19 | 0 | 2 |  | 17 | 20100222 | Mbita Island W | 8.75323 | 31.08457 | 85 | 105 | 17.7 | n/a |
| 22D6 | 40 | 40 | 30 | 0 |  | 10 | 20090522 | Ntilinga | 8.48139 | 30.46139 | 62 | 75 | n/a | m |
| 22D5 | 20 | 20 | 18 | 1 |  | 1 | 20090522 | Ntilinga | 8.48139 | 30.46139 | 69 | 84 | n/a | m |
| 22C3 | 5 | 5 | 2 | 1 |  | 2 | 20090522 | Ntilinga | 8.48139 | 30.46139 | 75 | 89 | n/a | m |
| 22C7 | 4 | 4 | 4 | 0 |  | 0 | 20090522 | Ntilinga | 8.48139 | 30.46139 | 83 | 100 | n/a | f |
| 22C5 | 12 | 12 | 7 | 0 |  | 5 | 20090522 | Ntilinga | 8.48139 | 30.46139 | 82 | 97 | n/a | f |
| 22D4 | 2 | 2 | 2 | 0 |  | 0 | 20090522 | Ntilinga | 8.48139 | 30.46139 | 78 | 94 | n/a | m |
| 22D2 | 21 | 21 | 17 | 0 |  | 4 | 20090522 | Ntilinga | 8.48139 | 30.46139 | 66 | 80 | n/a | m |
| 22C1 | 60 | 60 | 44 | 0 |  | 16 | 20090522 | Kalomo Point | 8.41972 | 30.46139 | 84 | 102 | n/a | f |
| 22C2 | 12 | 12 | 12 | 0 |  | 0 | 20090522 | Ntilinga | 8.48139 | 30.46139 | 83 | 100 | n/a | m |
| 22C4 | 7 | 7 | 2 | 0 |  | 5 | 20090522 | Ntilinga | 8.48139 | 30.46139 | 70 | 85 | n/a | m |
| 22C6 | 215 | 215 | 26 | 13 | 93 | 83 | 20090522 | Ntilinga | 8.48139 | 30.46139 | 88 | 107 | n/a | m |
| 22C8 | 6 | 6 | 5 | 0 |  | 1 | 20090522 | Ntilinga | 8.48139 | 30.46139 | 70 | 84 | n/a | m |
| 22C9 | 3 | 3 | 3 | 0 |  | 0 | 20090522 | Ntilinga | 8.48139 | 30.46139 | 67 | 80 | n/a | m |
| DFI4 | 4 | 4 | 0 | 4 |  | 0 | 20110930 | Mvuna Island | 7.44403 | 30.55035 | 96 | 114 | 23.3 | n/a |
| CYA8 | 66 | 28 | 27 | 1 |  | 0 | 20110925 | Izinga | 8.04971 | 30.89774 | 99 | 118 | 21.7 | n/a |
| DBD7 | 16 | 16 | 12 | 0 |  | 4 | 20110924 | Chizumba | 7.86119 | 30.78339 | 84 | 102 | 15.5 | n/a |
| DFA1 | 164 | 24 | 18 | 4 |  | 2 | 20110921 | Kasanga | 8.4379 | 31.14615 | 67 | 82 | 5.25 | n/a |
| DFG6 | 2 | 2 | 0 | 2 |  | 0 | 20110921 | Kipili | 7.44432 | 30.59225 | 89 | 106 | 16.8 | n/a |
| DFG4 | 24 | 22 | 14 | 0 |  | 8 | 20110921 | Kipili | 7.44432 | 30.59225 | 95 | 15 | 21.5 | n/a |
| DDG5 | 24 | 12 | 2 | 1 |  | 9 | 20110926 | Loazi North | 8.30584 | 31.03492 | 104 | 127 | 30 | n/a |
| DMH8 | 73 | 12 | 11 | 0 |  | 1 | 20110920 | Fulve rocks | 7.95526 | 30.82228 | 76 | 92 | 10.5 | n/a |
| DIF1 | 2 | 2 | 0 | 2 |  | 0 | 20110930 | Kachese | 8.48052 | 30.47749 | 81 | 97 | 11.3 | n/a |
| DIF4 | 22 | 22 | 11 | 0 |  | 11 | 20110930 | Kachese | 8.48052 | 30.47749 | 79 | 93 | 12.3 | n/a |
| DDG7 | 24 | 24 | 12 | 6 |  | 6 | 20110926 | Loazi North | 8.30584 | 31.03492 | 72 | 85 | 8.3 | n/a |
| DAE7 | 5 | 5 | 4 | 1 |  | 0 | 20110923 | Mukiringa | 7.66508 | 30.70775 | 87 | 106 | 13.5 | n/a |
| DAH4 | 69 | 19 | 13 | 3 | 35 | 3 | 20110924 | Mukiringa | 7.66508 | 30.70775 | 74 | 95 | 9.8 | n/a |
| 36A8 | 23 | 23 | 0 | 0 |  | 23 | 20100222 | Mbita Island W | 8.75323 | 31.08457 | 84 | 106 | 19 | n/a |
| DDE2 | 0 | 0 | 0 | 0 |  | 0 | 20110926 | Loazi South | 8.31555 | 31.03531 | 91 | 105 | 20 | n/a |
| 36H1 | 0 | 0 | 0 | 0 |  | 0 | 20100223 | Kasakalawe Lodge | 8.78107 | 31.09151 | 94 | 118 | 23.8 | n/a |

**Table S4. Transect Survey Results.**

Lists the counts of model and both mimics at different locations across the Southern part of Lake Tanganyika.

| **Locality** | **Date** | ***Model a*** | ***Model b*** | ***Mimic*** | **Coordinates SOUTH** | **Coordinates EAST** |
| --- | --- | --- | --- | --- | --- | --- |
| Kachese | 23/08/14 | 13 | 2 | 0 | 8.49041 | 30.47759 |
| Chitweshiba 1 | 19/08/14 | 26 | 2 | 0 | 8.59576 | 30.80801 |
| Chitweshiba 2 | 20/08/14 | 35 | 5 | 0 | 8.59576 | 30.80801 |
| Misepa | 20/08/14 | 12 | 1 | 5 | 8.58711 | 30.80176 |
| Kabwensolo | 18/08/14 | 27 | 3 | 4 | 8.60835 | 30.82778 |
| Misepa | 30/08/14 | 14 | 1 | 0 | 8.58711 | 30.80176 |
| Katete | 26/08/14 | 9 | 1 | 0 | 8.33879 | 30.50795 |

**Table S5. Results from the taxonomic assignment of individual scales**

*P. straeleni Ref. Nr.:* Reference number of *P. straeleni* stomach. *Scale DNA:* Reference number of scale. *BLAST result:* Taxonomic assignment using BLAST. *Phylo Results:* Taxonomic assignment using phylogenetic analyses. Empty rows: scales for which we could not retrieve a DNA sequence.

| *P. straeleni* Ref. Nr. | Scale DNA | *BLAST Result* | *Phylo Result* |
| --- | --- | --- | --- |
| 21D6 | 1 | *Eretmodus cyanostictus* | *Eretmodus cyanostictus* |
| 21D6 | 2 | *Eretmodus cyanostictus* | *Eretmodus cyanostictus* |
| 21D6 | 3 | *Eretmodus cyanostictus* | *Eretmodus cyanostictus* |
| 21D6 | 4 | *Aulonocranus dewindti* | *Ectodus descampsii* |
| 21D6 | 5 | *Eretmodus cyanostictus* | *Eretmodus cyanostictus* |
| 21D6 | 6 | *Aulonocranus dewindti* | *Aulonocranus dewindti* |
| 21D6 | 7 | *Eretmodus cyanostictus* | *Eretmodus cyanostictus* |
| 21D6 | 8 | *Ere. cya./Pet. fam.* | *Eretmodus cyanostictus* |
| 21D6 | 9 | *Eretmodus cyanostictus* | *Eretmodus cyanostictus* |
| 21D6 | 10 | *Eretmodus cyanostictus* | *Eretmodus cyanostictus* |
| 21D6 | 11 |  |  |
| 21D6 | 12 |  |  |
| 21D6 | 13 | *Eretmodus cyanostictus* | *Eretmodus cyanostictus* |
| 21D6 | 14 | *Eretmodus cyanostictus* | *Eretmodus cyanostictus* |
| 21D6 | 15 | *Aulonocranus dewindti* | *Aulonocranus dewindti* |
| 21D6 | 16 | *Eretmodus cyanostictus* | *Eretmodus cyanostictus* |
| 21D6 | 17 |  |  |
| 21D6 | 18 | *Eretmodus cyanostictus* | *Eretmodus cyanostictus* |
| 21D6 | 19 | *Petrochromis polyodon* | *Petrochromis polyodon* |
| 21D6 | 20 | *Eretmodus cyanostictus* | *Eretmodus cyanostictus* |
| 21D6 | 21 |  |  |
| 21D6 | 22 | *Ophthalmotilapia ventralis* | *Cyathopharynx furcifer* |
| 21D6 | 23 | *Eretmodus cyanostictus* | *Eretmodus cyanostictus* |
| 21D6 | 24 |  |  |
| 36A8 | 50 |  |  |
| 36A8 | 51 |  |  |
| 36A8 | 52 |  |  |
| 36A8 | 53 |  |  |
| 36A8 | 54 |  |  |
| 36A8 | 55 |  |  |
| 36A8 | 56 |  |  |
| 36A8 | 57 |  |  |
| 36A8 | 58 |  |  |
| 36A8 | 59 |  |  |
| 36A8 | 60 |  |  |
| 36A8 | 61 |  |  |
| 36A8 | 62 |  |  |
| 36A8 | 63 |  |  |
| 36A8 | 64 |  |  |
| 36A8 | 65 |  |  |
| 36A8 | 66 |  |  |
| 36A8 | 67 |  |  |
| 36A8 | 68 |  |  |
| 36A8 | 69 |  |  |
| 36A8 | 70 |  |  |
| 36A8 | 71 |  |  |
| 36A8 | 72 |  |  |
| 36A9 | 25 | *Tropheus sp.* | *Tropheus brichardi* |
| 36A9 | 26 | *Tropheus sp.* | *Tropheus brichardi* |
| 36A9 | 27 | *Petrochromis famula* | *Petrochromis famula* |
| 36A9 | 28 | *Tropheus sp.* | *Tropheus brichardi* |
| 36A9 | 29 | *Tropheus sp.* | *Tropheus brichardi* |
| 36A9 | 30 | *Julidochromis transcriptus* | *Julidochromis transcriptus* |
| 36A9 | 31 | *Aulonocranus dewindti* | *Aulonocranus dewindti* |
| 36A9 | 32 |  |  |
| 36A9 | 33 | *Julidochromis transcriptus* | *Julidochromis transcriptus* |
| 36A9 | 34 | *Callochromis macrops* | *Callochromis macrops* |
| 36A9 | 35 | *Jul. bri./Jul. dic./Cha. pop.* | *Neolamprologus christyi* |
| 36A9 | 36 | *Callochromis macrops* | *Callochromis macrops* |
| 36A9 | 37 |  |  |
| 36A9 | 38 | *Plecodus straeleni* | *Plecodus straeleni* |
| 36A9 | 39 | *Plecodus straeleni* | *Plecodus straeleni* |
| 36A9 | 40 | *Callochromis macrops* | *Callochromis macrops* |
| 36A9 | 41 | *Astatotilapia burtoni* | *Simochromis diagramma* |
| 36A9 | 42 |  |  |
| 36A9 | 43 | *Aulonocranus dewindti* | *Aulonocranus dewindti* |
| 36A9 | 44 | *Callochromis macrops* | *Callochromis macrops* |
| 36A9 | 45 |  |  |
| 36A9 | 46 | *Callochromis macrops* | *Callochromis macrops* |
| 36A9 | 47 | *Neolamprologus/Julidochromis* | *Neolamprologus christyi* |
| 36A9 | 48 | *Petrochromis polyodon* | *Pettex/Petpol/Peteph* |
| 36A9 | 49 |  |  |
| 36A9 | 73 | *Julidochromis transcriptus* | *Julidochromis transcriptus* |
| 36A9 | 74 | *Callochromis macrops* | *Callochromis macrops* |
| 36A9 | 75 |  |  |
| 36A9 | 76 | *Julidochromis transcriptus* | *Julidochromis transcriptus* |
| 36A9 | 77 | *Jul. dic./Neo. cyl./Tel. tem.* | *Julidochromis dickfeldi* |
| 36A9 | 78 | *Julidochromis transcriptus* | *Julidochromis transcriptus* |
| 36A9 | 79 | *Tropheus sp.* | *Tropheus brichardi* |
| 36A9 | 80 | *Julidochromis transcriptus* | *Julidochromis transcriptus* |
| 36A9 | 81 | *Julidochromis transcriptus* | *Julidochromis transcriptus* |
| 36A9 | 82 | *Neolamprologus tetracanthus* | *Neolamprologus modestus* |
| 36A9 | 83 |  |  |
| 36B1 | 188 | *Lobochilotes labiatus* | *Lobochilotes labiatus* |
| 36B1 | 189 | *Plecodus straeleni* | *Plecodus straeleni* |
| 36B1 | 190 | *Plecodus straeleni* | *Plecodus straeleni* |
| 36B1 | 191 | *Plecodus straeleni* | *Plecodus straeleni* |
| 36B1 | 192 | *Plecodus straeleni* | *Plecodus straeleni* |
| 36B1 | 193 |  |  |
| 36B1 | 194 |  |  |
| 36B1 | 195 |  |  |
| 36A7 | 198 |  |  |
| 36A7 | 199 |  |  |
| 36A7 | 200 |  |  |
| 36A7 | 201 |  |  |
| 36A7 | 202 |  |  |
| 36A7 | 203 |  |  |
| 36A7 | 204 |  |  |
| 36A7 | 205 |  |  |
| 36A7 | 206 |  |  |
| 36A7 | 207 |  |  |
| 36A7 | 208 |  |  |
| 36A7 | 209 |  |  |
| 36A7 | 210 |  |  |
| 36A7 | 211 |  |  |
| 36A7 | 212 |  |  |
| 36A7 | 213 |  |  |
| 36A7 | 214 |  |  |
| 36A7 | 215 | *Plecodus straeleni* | *Plecodus straeleni* |
| 36A7 | 216 | *Plecodus straeleni* | *Plecodus straeleni* |
| 48B9 | 84 | *Tropheus sp.* | *Tropheus brichardi* |
| 48B9 | 85 | *Petrochromis polyodon* | *Lobochilotes labiatus* |
| 48B9 | 86 | *Simochromis diagramma* | *Lobochilotes labiatus* |
| 48B9 | 87 | *Petrochromis polyodon* | *Lobochilotes labiatus* |
| 48B9 | 88 | *Petrochromis polyodon* | *Petrochromis polyodon* |
| 48B9 | 89 | *Petrochromis sp.* | *Petrochromis sp. Texas* |
| 48B9 | 90 | *Plestr/Permic* | *Plestr/Permic* |
| 48B9 | 91 | *Petrochromis sp.* | *Lobochilotes labiatus* |
| 48B9 | 92 | *Plestr/Permic* | *Plecodus straeleni* |
| 48B9 | 93 | *Petrochromis polyodon* | *Petrochromis polyodon* |
| 48B9 | 94 | *Petrochromis polyodon* | *Pettex/Petpol/Peteph* |
| 48B9 | 95 | *Cyphotilapia gibberosa* | *Cyphotilapia gibberosa* |
| 48B9 | 96 | *Plestr/Permic* | *Plestr/Permic* |
| 36H4 | 152 | *Perissodus microlepis* | *Plecodus straeleni* |
| 36H4 | 153 | *Oph. het./Cya. fur./Cun. lon.* | *Cyafur/Ophhet/Ophnas* |
| 36H4 | 154 |  |  |
| 36H4 | 155 |  |  |
| 36H4 | 156 | *Plecodus straeleni* | *Plecodus straeleni* |
| 36H4 | 157 |  |  |
| 36H4 | 158 |  |  |
| 36H4 | 159 |  |  |
| 36H4 | 160 |  |  |
| 36H4 | 161 | *Plecodus straeleni* | *Plecodus straeleni* |
| 36H4 | 162 | *Oph. het/ven./Per. mic.* | *Unknown* |
| 36H4 | 163 | *Aulonocranus dewindti* | *Aulonocranus dewindti* |
| 36H4 | 164 | *Plecodus straeleni* | *Plecodus straeleni* |
| 36H4 | 165 | *Aulonocranus dewindti* | *Aulonocranus dewindti* |
| 36H4 | 166 | *Plecodus straeleni* | *Plecodus straeleni* |
| 36H4 | 167 | *Plecodus straeleni* | *Plecodus straeleni* |
| 36H4 | 168 | *Plecodus straeleni* | *Plecodus straeleni* |
| 36H4 | 169 | *Aulonocranus dewindti* | *Aulonocranus dewindti* |
| 36H4 | 170 | *Cunningtonia longiventralis* | *Lesper/Cunlon/Auldew* |
| 36H4 | 171 | *Ophthalmotilapia heterodonta* | *Ectodini* |
| 36H4 | 172 | *Aulonocranus dewindti* | *Aulonocranus dewindti* |
| 36H4 | 173 | *Ophthalmotilapia ventralis* | *Ophthalmotilapia ventralis* |
| 36H4 | 174 |  |  |
| 36H4 | 175 | *Plecodus straeleni* | *Plecodus straeleni* |
| 36H4 | 176 | *Ophthalmotilapia ventralis* | *Ophthalmotilapia ventralis* |
| 36H4 | 177 | *Aulonocranus dewindti* | *Aulonocranus dewindti* |
| 36H4 | 178 | *Ophthalmotilapia heterodonta* | *Cyafur/Ophhet/Ophnas* |
| 36H4 | 179 | *Aulonocranus dewindti* | *Aulonocranus dewindti* |
| 36H4 | 180 | *Ophthalmotilapia ventralis* | *Ophthalmotilapia ventralis* |
| 36H4 | 181 | *Ophthalmotilapia ventralis* | *Ophhet/Ophnas* |
| 36H4 | 182 | *Aulonocranus dewindti* | *Aulonocranus dewindti* |
| 36H4 | 183 | *Aulonocranus dewindti* | *Aulonocranus dewindti* |
| 36H4 | 184 | *Aulonocranus dewindti* | *Aulonocranus dewindti* |
| 36H4 | 185 | *Aulonocranus dewindti* | *Aulonocranus dewindti* |
| 36H4 | 186 | *Ophthalmotilapia ventralis* | *Cyafur/Ophhet/Ophnas* |
| 36H4 | 187 | *Ophthalmotilapia ventralis* | *Cyafur/Ophhet/Ophnas* |
| 36H2 | 109 | *Lamprologus lemairii* | *UNKNOWN CICHLID* |
| 36H2 | 110 |  |  |
| 36H2 | 111 | *Lamprologus lemairii* | *Lamprologus lemairii* |
| 36H2 | 112 | *Aulonocranus dewindti* | *Aulonocranus dewindti* |
| 36H2 | 113 | *Lamprologus lemairii* | *Lamprologus lemairii* |
| 36H2 | 114 | *Lamprologus lemairii* | *Lamprologus lemairii* |
| 36H2 | 115 | *Oph. het./Cya. fur./Cun. lon.* | *Cyafur/Ophhet/Ophnas* |
| 36H2 | 116 | *Lamprologus lemairii* | *Lamprologus lemairii* |
| 36H2 | 117 | *Lamprologus lemairii* | *Lamprologus lemairii* |
| 36H2 | 118 | *Lamprologus lemairii* | *Lamprologus lemairii* |
| 36H2 | 119 | *Lamprologus lemairii* | *Lamprologus lemairii* |
| 36H2 | 120 | *Oph. het./Cya. fur./Cun. lon.* | *Cyafur/Ophhet/Ophnas* |
| 36H2 | 121 | *Oph. het./Cya. fur./Oph. ven.* | *Ectodini* |
| 36H2 | 122 | *Lamprologus lemairii* | *Neolamprologus leloupi* |
| 36H2 | 123 | *Petrochromis famula* | *Petrochromis famula* |
| 36H2 | 124 | *Petrochromis famula* | *Petrochromis famula* |
| 36H2 | 125 |  |  |
| 36H2 | 126 | *Cya. fur./Oph. ven.* | *Cyafur/Ophhet/Ophnas* |
| 36H2 | 127 | *Cya. fur./Oph. ven.* | *Cyafur/Ophhet/Ophnas* |
| 36H2 | 128 | *Plecodus straeleni* | *Plecodus straeleni* |
| 36H2 | 129 | *Oph. het./Cya. fur./Oph. ven.* | *Cyafur/Ophhet/Ophnas* |
| 36H2 | 130 | *Simmochromis diagramma* | *Simdia/Simbab/Simple* |
| 36H2 | 131 | *Oph. het./Cya. fur./Oph. ven.* | *Cyafur/Ophhet/Ophnas* |
| 36H2 | 132 | *Oph. ven./Oph. het./Cya. fiur.* | *Ectodini* |
| 36H2 | 133 | *Cya. fur./Oph. het./Oph. ven.* | *Cyafur/Ophhet/Ophnas* |
| 36H2 | 134 | *Cya. fur./Oph. het./Oph. ven.* | *Cyafur/Ophhet/Ophnas* |
| 36H2 | 135 | *Cya. fur./Oph. het./Oph. ven.* | *Cyafur/Ophhet/Ophnas* |
| 36H2 | 136 | *Cya. fur./Oph. het./Oph. ven.* | *Cyafur/Ophhet/Ophnas* |
| 36H2 | 137 | *Aulonocranus dewindti* | *Aulonocranus dewindti* |
| 36H2 | 138 | *Plecodus straeleni* | *Perissodini* |
| 36H2 | 139 | *Lamprologus lemairii* | *Ectodini* |
| 36H2 | 140 | *Cya. fur./Oph. het./Oph. ven.* | *Cyafur/Ophhet/Ophnas* |
| 36H2 | 141 | *Cya. fur./Oph. het./Oph. ven.* | *Cyafur/Ophhet/Ophnas* |
| 36H2 | 142 | *Petrochromis famula* | *Petrochromis famula* |
| 36H2 | 143 | *Cya. fur./Oph. het./Oph. ven.* | *Cyafur/Ophhet/Ophnas* |
| 36H2 | 144 | *Cya. fur./Oph. het./Oph. ven.* | *Cyafur/Ophhet/Ophnas* |
| 36H2 | 145 |  |  |
| 36H2 | 146 | *Petrochromis famula* | *Petrochromis famula* |
| 36H2 | 147 | *Aulonocranus dewindti* | *Aulonocranus dewindti* |
| 36H2 | 148 | *Astatotilapia burtoni* | *UNKNOWN CICHLID* |
| 36H2 | 149 |  |  |
| 36H2 | 150 | *Cya. fur./Oph. ven./Oph. het.* | *Cyathopharynx furcifer* |
| 36H2 | 151 |  |  |
| 36H3 | 196 | *Plecodus straeleni* | *Plestr/Permic* |
| 36H3 | 197 | *Lamprologus lemairii* | *Lamprologus lemairii* |
| DFI4 | 624 | *Plecodus straeleni* | *Plestr/Permic* |
| DFI4 | 625 | *Perissodus microlepis* | *Perissodus microlepis* |
| DFI4 | 627 | *Perissodus microlepis* | *Perissodus microlepis* |
| DFI4 | 628 | *Plecodus straeleni* | *Perissodus microlepis* |
| 22C1 | 321 | *Haplotaxodon microlepis* | *Haplotaxodon microlepis* |
| 22C1 | 322 | *Haplotaxodon microlepis* | *Haplotaxodon microlepis* |
| 22C1 | 323 | *Haplotaxodon microlepis* | *Haplotaxodon microlepis* |
| 22C1 | 324 |  |  |
| 22C1 | 325 |  |  |
| 22C1 | 326 |  |  |
| 22C1 | 327 |  |  |
| 22C1 | 328 |  |  |
| 22C1 | 329 | *Lepidiolamprologus sp.* | *Lepidiolamprologus profundicola* |
| 22C1 | 330 |  |  |
| 22C1 | 331 | *Petrochromis fasciolatus* | *Petfas/Petort/Intloc* |
| 22C1 | 332 | *Haplotaxodon microlepis* | *Haplotaxodon microlepis* |
| 22C1 | 333 | *Haplotaxodon microlepis* | *Haplotaxodon microlepis* |
| 22C1 | 334 | *Haplotaxodon microlepis* | *Haplotaxodon microlepis* |
| 22C1 | 335 |  |  |
| 22C1 | 336 | *Haplotaxodon microlepis* | *Haplotaxodon microlepis* |
| 22C1 | 337 | *Haplotaxodon microlepis* | *Haplotaxodon microlepis* |
| 22C1 | 338 | *Haplotaxodon microlepis* | *Haplotaxodon microlepis* |
| 22C1 | 339 | *Haplotaxodon microlepis* | *Haplotaxodon microlepis* |
| 22C1 | 340 | *Haplotaxodon microlepis* | *Haplotaxodon microlepis* |
| 22C1 | 341 | *Lep. sp. 'Zambia yellow'* | *Lepidiolamprologus profundicola* |
| 22C1 | 342 | *Lep. sp. 'Zambia yellow'* | *Lepidiolamprologus profundicola* |
| 22C1 | 343 | *Haplotaxodon microlepis* | *Haplotaxodon microlepis* |
| 22C1 | 344 | *Tro. dub./Perciformes sp.* |  |
| 22C1 | 345 |  |  |
| 22C1 | 346 | *Lep. sp. 'Zambia yellow'* | *Lepidiolamprologus profundicola* |
| 22C1 | 347 | *Petrochromis polyodon* | *Petpol/Peteph* |
| 22C1 | 348 | *Lep. sp. 'Zambia yellow'* | *Lepidiolamprologus profundicola* |
| 22C1 | 349 | *Lep. sp. 'Zambia yellow'* | *Lepidiolamprologus profundicola* |
| 22C1 | 350 | *Haplotaxodon microlepis* | *Haplotaxodon microlepis* |
| 22C1 | 351 | *Haplotaxodon microlepis* | *Haplotaxodon microlepis* |
| 22C1 | 352 | *Haplotaxodon microlepis* | *Haplotaxodon microlepis* |
| 22C1 | 353 | *Haplotaxodon microlepis* | *Haplotaxodon microlepis* |
| 22C1 | 354 |  |  |
| 22C1 | 355 | *Haplotaxodon microlepis* | *Haplotaxodon microlepis* |
| 22C1 | 356 | *Haplotaxodon microlepis* | *Haplotaxodon microlepis* |
| 22C1 | 357 |  |  |
| 22C1 | 358 | *Lep. sp. 'Zambia yellow'* | *Lepidiolamprologus profundicola* |
| 22C1 | 359 | *Haplotaxodon microlepis* | *Haplotaxodon microlepis* |
| 22C1 | 360 | *Haplotaxodon microlepis* | *Haplotaxodon microlepis* |
| 22C1 | 361 | *Haplotaxodon microlepis* | *Haplotaxodon microlepis* |
| 22C1 | 362 | *Haplotaxodon microlepis* | *Haplotaxodon microlepis* |
| 22C1 | 363 | *Lep. sp. 'Zambia yellow'* | *Lepidiolamprologus profundicola* |
| 22C1 | 364 | *Lep. sp. 'Zambia yellow'* | *Lepidiolamprologus profundicola* |
| 22C1 | 365 | *Lep. sp. 'Zambia yellow'* | *Lepidiolamprologus profundicola* |
| 22C1 | 366 |  |  |
| 22C1 | 367 | *Tropheus duboisi* | *Haplotaxodon microlepis* |
| 22C1 | 368 |  |  |
| 22C1 | 369 | *Tropheus duboisi* | *Haplotaxodon microlepis* |
| 22C1 | 370 | *Tropheus duboisi* | *Haplotaxodon microlepis* |
| 22C1 | 371 | *Lep. sp. 'Zambia yellow'* | *Lepidiolamprologus profundicola* |
| 22C1 | 372 | *Lep. sp. 'Zambia yellow'* | *Lepidiolamprologus profundicola* |
| 22C1 | 373 |  |  |
| 22C1 | 374 | *Haplotaxodon microlepis* | *Haplotaxodon microlepis* |
| 22C1 | 375 | *Haplotaxodon microlepis* | *Haplotaxodon microlepis* |
| 22C1 | 376 |  |  |
| 22C1 | 377 | *Petrochromis fasciolatus* | *Intloc/Petort* |
| 22C1 | 378 | *Haplotaxodon microlepis* | *Haplotaxodon microlepis* |
| 22C1 | 379 | *Haplotaxodon microlepis* | *Haplotaxodon microlepis* |
| 22C1 | 380 |  |  |
| 22C2 | 381 | *Tel. dho./Neo. sav.* | *Telmatochromis sp.* |
| 22C2 | 382 | *Petrochromis polyodon* | *Petrochromis polyodon* |
| 22C2 | 383 | *Lep. sp. 'Zambia yellow'* | *Lepidiolamprologus profundicola* |
| 22C2 | 384 | *Lobochilotes labiatus* | *Petrochromis trewavasae* |
| 22C2 | 385 | *Boulengerochromis microlepis* | *Boulengerochromis microlepis* |
| 22C2 | 386 | *Neo. tet./Neo. mod.* | *Neolamprologus modestus* |
| 22C2 | 387 | *Neolamprologus multifasciatus* | *Lamprologini* |
| 22C2 | 388 | *Haplotaxodon microlepis* | *Haplotaxodon microlepis* |
| 22C2 | 389 | *Petrochromis polyodon* | *Petrochromis polyodon* |
| 22C2 | 390 | *Sim. dia./Lob. lab.* | *Intloc/Petort* |
| 22C2 | 391 | *Boulengerochromis microlepis* | *Boulengerochromis microlepis* |
| 22C2 | 392 | *Boulengerochromis microlepis* | *Boulengerochromis microlepis* |
| 22C3 | 256 |  |  |
| 22C3 | 257 | *Neo. pet./Neo. leu.* | *Neolamprologus petricola* |
| 22C3 | 258 | *Neolamprologus petricola* | *Neolamprologus petricola* |
| 22C3 | 259 |  |  |
| 22C3 | 273 | *Plecodus straeleni* | *Plestr/Permic* |
| 22C4 | 393 |  |  |
| 22C4 | 394 |  |  |
| 22C4 | 395 |  |  |
| 22C4 | 396 |  |  |
| 22C4 | 397 | *Petrochromis polyodon* | *Petrochromis polyodon* |
| 22C4 | 398 |  |  |
| 22C4 | 399 | *Alt. com./Lep. elo.* | *UNKNOWN CICHLID* |
| 22C5 | 263 |  |  |
| 22C5 | 264 | *Tropheus sp./Kachee/moori* | *Tropheus moori* |
| 22C5 | 265 |  |  |
| 22C5 | 266 |  |  |
| 22C5 | 267 | *Petrochromis polyodon* | *Petrochromis polyodon* |
| 22C5 | 268 | *Tropheus sp./Kachee/moori* | *Tropheus moori* |
| 22C5 | 269 | *Petrochromis polyodon* | *Petrochromis polyodon* |
| 22C5 | 270 |  |  |
| 22C5 | 271 |  |  |
| 22C5 | 272 | *Tel. dho./Neo. sav.* | *Telmatochromis temporalis* |
| 22C5 | 275 | *Petrochromis polyodon* | *Petrochromis trewavasae* |
| 22C5 | 276 | *Petrochromis polyodon* | *Petrochromis polyodon* |
| 22C6 | 400 |  |  |
| 22C6 | 401 |  |  |
| 22C6 | 402 |  |  |
| 22C6 | 403 |  |  |
| 22C6 | 404 |  |  |
| 22C6 | 405 | *Plecodus straeleni* | *Plestr/Permic* |
| 22C6 | 406 | *Neolamprologus prochilus* | *Neolamprologus obscurus* |
| 22C6 | 407 |  |  |
| 22C6 | 408 |  |  |
| 22C6 | 409 |  |  |
| 22C6 | 410 | *Mastacembelus ellipsifer* |  |
| 22C6 | 411 | *Lepidiolamprologus sp.* | *Lepidiolamprologus profundicola* |
| 22C6 | 412 | *Mastacembelus ellipsifer* |  |
| 22C6 | 413 | *Lamprologus callipterus* | *Lamprologus callipterus* |
| 22C6 | 414 | *Mastacembelus ellipsifer* |  |
| 22C6 | 415 | *Mastacembelus ellipsifer* |  |
| 22C6 | 416 |  |  |
| 22C6 | 417 |  |  |
| 22C6 | 418 |  |  |
| 22C6 | 419 | *Perissodus microlepis* | *Ophhet/Ophnas* |
| 22C6 | 420 | *Tel. dho./Neo. sav./Neo. pro.* | *Neolamprologus prochilus/obscurus* |
| 22C6 | 421 | *Lep. sp. 'Zambia yellow'* | *Lepidiolamprologus profundicola* |
| 22C6 | 422 | *Astatotilapia burtoni* | *Astatotilapia burtoni* |
| 22C6 | 423 | *Petrochromis polyodon* | *Petrochromis polyodon* |
| 22C6 | 424 | *Perissodus microlepis/Ple. str.* | *Plecodus straeleni* |
| 22C6 | 425 |  |  |
| 22C6 | 429 | *Perissodus microlepis/Ple. str.* | *Plecodus straeleni* |
| 22C6 | 430 | *Plecodus straeleni* | *Plestr/Permic* |
| 22C6 | 431 |  |  |
| 22C6 | 432 |  |  |
| 22C6 | 433 |  |  |
| 22C6 | 435 |  |  |
| 22C6 | 441 |  |  |
| 22C6 | 442 | *Ophthalmotilapia nasuta* | *Ophthalmotilapia nasuta* |
| 22C6 | 443 | *Petrochromis polyodon* | *Petrochromis polyodon* |
| 22C6 | 444 | *Ophthalmotilapia nasuta* | *Ophthalmotilapia nasuta* |
| 22C6 | 445 | *Ophthalmotilapia nasuta* | *Ophthalmotilapia nasuta* |
| 22C6 | 446 | *Petrochromis polyodon* | *Petrochromis polyodon* |
| 22C6 | 447 | *Ophthalmotilapia nasuta* | *Ophthalmotilapia nasuta* |
| 22C6 | 448 | *Ophthalmotilapia nasuta* | *Ophthalmotilapia nasuta* |
| 22C6 | 449 | *Ophthalmotilapia nasuta* | *Ophthalmotilapia nasuta* |
| 22C6 | 450 | *Ophthalmotilapia nasuta* | *Ophthalmotilapia nasuta* |
| 22C6 | 451 | *Petrochromis polyodon* | *Tropheus sp.* |
| 22C6 | 452 | *Petrochromis polyodon* | *Petpol/Peteph* |
| 22C6 | 453 | *Mastacembelus ellipsifer* |  |
| 22C6 | 454 |  |  |
| 22C6 | 455 |  |  |
| 22C6 | 456 |  |  |
| 22C6 | 457 | *Plecodus straeleni* | *Plecodus straeleni* |
| 22C6 | 458 | *Plecodus straeleni* | *Plestr/Permic* |
| 22C6 | 459 | *Mastacembelus ellipsifer* |  |
| 22C6 | 460 | *Plecodus straeleni* | *Perissodini* |
| 22C6 | 461 | *Mastacembelus ellipsifer* |  |
| 22C6 | 462 |  |  |
| 22C6 | 463 |  |  |
| 22C6 | 464 | *Mastacembelus ellipsifer* |  |
| 22C6 | 465 | *Perissodus microlepis/Ple. str.* | *Perissodini* |
| 22C6 | 466 | *Gram. lem./Cal. ple.* | *UNKNOWN CICHLID* |
| 22C6 | 467 | *Gram. lem./Cal. ple.* | *UNKNOWN CICHLID* |
| 22C6 | 468 | *Gram. lem./Cal. ple.* | *UNKNOWN CICHLID* |
| 22C6 | 469 | *Mastacembelus ellipsifer* | *UNKNOWN CICHLID* |
| 22C6 | 470 | *Mastacembelus ellipsifer* | *Perissodini* |
| 22C6 | 471 | *Mastacembelus ellipsifer* | *UNKNOWN CICHLID* |
| 22C6 | 472 | *Mastacembelus ellipsifer* | *Perissodini* |
| 22C6 | 473 | *Perissodus microlepis/Ple. str.* | *Astatotilapia burtoni* |
| 22C6 | 474 | *Gram. lem./Cal. ple.* | *UNKNOWN CICHLID* |
| 22C6 | 475 |  |  |
| 22C6 | 476 | *Perissodus microlepis/Ple. str.* | *Plestr/Permic* |
| 22C6 | 477 | *Mastacembelus ellipsifer* |  |
| 22C6 | 478 | *Mastacembelus ellipsifer* |  |
| 22C6 | 479 |  |  |
| 22C6 | 480 |  |  |
| 22C6 | 481 | *Plecodus straeleni* | *Plestr/Permic* |
| 22C6 | 482 | *Perissodus microlepis/Ple. str.* | *Plecodus straeleni* |
| 22C6 | 483 |  |  |
| 22C6 | 484 |  |  |
| 22C6 | 485 |  |  |
| 22C6 | 486 | *Perissodus microlepis/Ple. str.* | *Plestr/Permic* |
| 22C6 | 487 |  |  |
| 22C6 | 488 | *Mastacembelus ellipsifer* |  |
| 22C6 | 489 | *Mastacembelus ellipsifer* |  |
| 22C6 | 490 |  |  |
| 22C6 | 491 | *Mastacembelus ellipsifer* |  |
| 22C6 | 492 | *Mastacembelus ellipsifer* |  |
| 22C6 | 493 |  |  |
| 22C6 | 494 |  |  |
| 22C6 | 495 |  |  |
| 22C6 | 496 |  |  |
| 22C6 | 497 | *Mastacembelus ellipsifer* |  |
| 22C6 | 498 |  |  |
| 22C6 | 499 |  |  |
| 22C6 | 500 |  |  |
| 22C6 | 501 |  |  |
| 22C6 | 502 |  |  |
| 22C6 | 503 |  |  |
| 22C6 | 504 |  |  |
| 22C6 | 505 | *Mastacembelus ellipsifer* |  |
| 22C6 | 506 |  |  |
| 22C6 | 507 | *Mastacembelus ellipsifer* |  |
| 22C6 | 508 | *Mastacembelus ellipsifer* |  |
| 22C6 | 509 | *Mastacembelus ellipsifer* |  |
| 22C6 | 510 |  |  |
| 22C6 | 511 |  |  |
| 22C6 | 512 |  |  |
| 22C6 | 513 |  |  |
| 22C6 | 514 | *Mastacembelus ellipsifer* |  |
| 22C6 | 515 | *Mastacembelus ellipsifer* |  |
| 22C6 | 516 | *Mastacembelus ellipsifer* |  |
| 22C6 | 517 | *Mastacembelus ellipsifer* |  |
| 22C6 | 518 |  |  |
| 22C6 | 519 | *Mastacembelus ellipsifer* |  |
| 22C6 | 520 |  |  |
| 22C6 | 521 |  |  |
| 22C6 | 522 |  |  |
| 22C6 | 523 | *Mastacembelus ellipsifer* |  |
| 22C6 | 524 | *Mastacembelus ellipsifer* |  |
| 22C6 | 525 |  |  |
| 22C6 | 526 |  |  |
| 22C6 | 527 |  |  |
| 22C6 | 528 |  |  |
| 22C6 | 529 |  |  |
| 22C6 | 530 | *Mastacembelus ellipsifer* |  |
| 22C6 | 531 | *Mastacembelus ellipsifer* |  |
| 22C6 | 532 | *Mastacembelus ellipsifer* |  |
| 22C6 | 533 | *Mastacembelus ellipsifer* |  |
| 22C6 | 534 | *Mastacembelus ellipsifer* |  |
| 22C6 | 535 |  |  |
| 22C6 | 536 |  |  |
| 22C6 | 537 | *Mastacembelus ellipsifer* |  |
| 22C6 | 538 | *Mastacembelus ellipsifer* |  |
| 22C6 | 539 | *Mastacembelus ellipsifer* |  |
| 22C6 | 540 | *Mastacembelus ellipsifer* |  |
| 22C6 | 541 |  |  |
| 22C6 | 542 | *Mastacembelus ellipsifer* |  |
| 22C6 | 543 | *Mastacembelus ellipsifer* |  |
| 22C6 | 544 |  |  |
| 22C6 | 545 |  |  |
| 22C6 | 546 | *Mastacembelus ellipsifer* |  |
| 22C6 | 547 | *Mastacembelus ellipsifer* |  |
| 22C6 | 548 | *Mastacembelus ellipsifer* |  |
| 22C6 | 549 | *Mastacembelus ellipsifer* |  |
| 22C6 | 550 |  |  |
| 22C6 | 551 | *Mastacembelus ellipsifer* |  |
| 22C6 | 552 | *Mastacembelus ellipsifer* |  |
| 22C6 | 553 | *Mastacembelus ellipsifer* |  |
| 22C6 | 554 | *Mastacembelus ellipsifer* |  |
| 22C6 | 555 | *Mastacembelus ellipsifer* |  |
| 22C6 | 556 | *Mastacembelus ellipsifer* |  |
| 22C6 | 557 | *Mastacembelus ellipsifer* |  |
| 22C6 | 558 |  |  |
| 22C6 | 559 |  |  |
| 22C6 | 560 |  |  |
| 22C6 | 561 |  |  |
| 22C6 | 562 | *Mastacembelus ellipsifer* |  |
| 22C6 | 563 | *Mastacembelus ellipsifer* |  |
| 22C6 | 564 | *Mastacembelus ellipsifer* |  |
| 22C6 | 565 | *Mastacembelus ellipsifer* |  |
| 22C6 | 566 | *Mastacembelus ellipsifer* |  |
| 22C6 | 567 | *Mastacembelus ellipsifer* |  |
| 22C6 | 568 |  |  |
| 22C6 | 569 |  |  |
| 22C6 | 570 |  |  |
| 22C6 | 571 | *Mastacembelus ellipsifer* |  |
| 22C6 | 572 | *Mastacembelus ellipsifer* |  |
| 22C6 | 573 | *Mastacembelus ellipsifer* |  |
| 22C6 | 574 | *Mastacembelus ellipsifer* |  |
| 22C6 | 575 | *Mastacembelus ellipsifer* |  |
| 22C6 | 576 | *Mastacembelus ellipsifer* |  |
| 22C6 | 577 |  |  |
| 22C6 | 578 | *Mastacembelus ellipsifer* |  |
| 22C6 | 579 |  |  |
| 22C6 | 580 | *Mastacembelus ellipsifer* |  |
| 22C6 | 581 |  |  |
| 22C6 | 582 | *Mastacembelus ellipsifer* |  |
| 22C6 | 583 | *Mastacembelus ellipsifer* |  |
| 22C6 | 584 | *Mastacembelus ellipsifer* |  |
| 22C6 | 585 | *Mastacembelus ellipsifer* |  |
| 22C6 | 586 | *Mastacembelus ellipsifer* |  |
| 22C6 | 587 |  |  |
| 22C6 | 588 | *Mastacembelus ellipsifer* |  |
| 22C6 | 589 | *Mastacembelus ellipsifer* |  |
| 22C6 | 590 |  |  |
| 22C6 | 591 |  |  |
| 22C6 | 592 | *Mastacembelus ellipsifer* |  |
| 22C6 | 593 |  |  |
| 22C6 | 594 | *Mastacembelus ellipsifer* |  |
| 22C6 | 595 |  |  |
| 22C6 | 596 | *Mastacembelus ellipsifer* |  |
| 22C6 | 597 | *Mastacembelus ellipsifer* |  |
| 22C6 | 598 |  |  |
| 22C6 | 599 |  |  |
| 22C6 | 600 | *Mastacembelus ellipsifer* |  |
| 22C6 | 601 | *Mastacembelus ellipsifer* |  |
| 22C6 | 602 | *Mastacembelus ellipsifer* |  |
| 22C6 | 603 |  |  |
| 22C6 | 604 | *Mastacembelus ellipsifer* |  |
| 22C6 | 605 |  |  |
| 22C6 | 606 | *Mastacembelus ellipsifer* |  |
| 22C6 | 607 |  |  |
| 22C6 | 608 | *Mastacembelus ellipsifer* |  |
| 22C6 | 609 |  |  |
| 22C6 | 610 | *Mastacembelus ellipsifer* |  |
| 22C6 | 611 | *Mastacembelus ellipsifer* |  |
| 22C6 | 612 | *Mastacembelus ellipsifer* |  |
| 22C6 | 613 |  |  |
| 22C6 | 614 | *Mastacembelus ellipsifer* |  |
| 22C6 | 615 | *Mastacembelus ellipsifer* |  |
| 22C6 | 616 | *Mastacembelus ellipsifer* |  |
| 22C6 | 617 | *Mastacembelus ellipsifer* |  |
| 22C6 | 618 | *Mastacembelus ellipsifer* |  |
| 22C6 | 619 | *Mastacembelus ellipsifer* |  |
| 22C6 | 620 | *Mastacembelus ellipsifer* |  |
| 22C6 | 621 |  |  |
| 22C6 | 622 |  |  |
| 22C6 | 623 |  |  |
| 22C7 | 260 | *Petrochromis polyodon* | *Petrochromis polyodon* |
| 22C7 | 261 | *Petrochromis polyodon* | *Petrochromis polyodon* |
| 22C7 | 262 | *Petrochromis polyodon* | *Petrochromis polyodon* |
| 22C7 | 274 | *Petrochromis polyodon* | *Petrochromis polyodon* |
| 22C8 | 434 | *Neolamprologus furcifer* | *Neolamprologus furcifer* |
| 22C8 | 436 | *Petrochromis sp.* | *Petrochromis polyodon* |
| 22C8 | 437 |  |  |
| 22C8 | 438 | *Altolamprologus sp.* | *Altolamprologus sp.* |
| 22C8 | 439 | *Cunningtonia longiventralis* | *Aulonocranus dewindti* |
| 22C8 | 440 | *Altolamprologus sp.* | *Altolamprologus compressiceps* |
| 22C9 | 426 | *Petrochromis polyodon* | *Petrochromis polyodon* |
| 22C9 | 427 | *Tropheus sp.* | *Tropheus polli* |
| 22C9 | 428 | *Petrochromis polyodon* | *Tropheus sp.* |
| 22D2 | 300 | *Petrochromis polyodon* | *Petrochromis polyodon* |
| 22D2 | 301 |  |  |
| 22D2 | 302 | *Ast. bur./Tropheus sp./Oph. ven.* | *Petrochromis sp. Katete* |
| 22D2 | 303 | *Petrochromis polyodon* | *Petrochromis polyodon* |
| 22D2 | 304 | *Petrochromis polyodon* | *Petpol/Peteph* |
| 22D2 | 305 | *Lamprologus callipterus* | *Lamprologus callipterus* |
| 22D2 | 306 | *Tropheus sp.* | *Tropheus sp.* |
| 22D2 | 307 | *Oph. nas./Cya. fur./Oph. het.* | *Cyafur/Ophhet/Ophnas* |
| 22D2 | 308 | *Petrochromis polyodon* | *Petrochromis polyodon* |
| 22D2 | 309 | *Petrochromis polyodon* | *Petrochromis sp./Loblab* |
| 22D2 | 310 | *Petrochromis polyodon* | *Petrochromis polyodon* |
| 22D2 | 311 |  |  |
| 22D2 | 312 | *Oph. nas./Cya. fur./Oph. het.* | *Ophhet/Ophnas* |
| 22D2 | 313 | *Petrochromis polyodon* | *Petrochromis polyodon* |
| 22D2 | 314 | *Petrochromis polyodon* | *Petrochromis polyodon* |
| 22D2 | 315 |  |  |
| 22D2 | 316 | *Interochromis loocki* | *Petrochromis sp. Katete* |
| 22D2 | 317 | *Petrochromis polyodon* | *Petrochromis polyodon* |
| 22D2 | 318 | *Xenotilapia batyphila* | *Xenotilapia bathyphila* |
| 22D2 | 319 | *Ophthalmotilapia nasuta* | *Ophhet/Ophnas* |
| 22D2 | 320 | *Perciformes sp. BOLD/Tro. dub.* |  |
| 22D4 | 277 | *Oph. het./Cya. fur./Cun. lon.* | *Cyafur/Ophhet/Ophnas* |
| 22D4 | 278 | *Oph. het./Cya. fur./Cun. lon.* | *Cyafur/Ophhet/Ophnas* |
| 22D5 | 249 | *Neo. tet./Neo. mod.* | *Neolamprologus modestus* |
| 22D5 | 250 |  |  |
| 22D5 | 251 | *Neo. tet./Neo. mod.* | *Neolamprologus modestus* |
| 22D5 | 252 | *Neo. tet./Neo. mod.* | *Neolamprologus modestus* |
| 22D5 | 253 | *Neo. tet./Neo. mod.* | *Neolamprologus modestus* |
| 22D5 | 254 | *Neo. tet./Neo. mod.* | *Neolamprologus modestus* |
| 22D5 | 255 | *Neo. pet./Neo. leu.* | *Neolamprologus petricola* |
| 22D5 | 279 | *Lam. call./Neo. cau./Lep. elo.* | *Neolamprologus modestus* |
| 22D5 | 280 | *Neo. tet./Neo. mod.* | *Neolamprologus modestus* |
| 22D5 | 281 | *Neo. tet./Neo. mod.* | *Neolamprologus modestus* |
| 22D5 | 282 | *Neo. tet./Neo. mod.* | *Neolamprologus modestus* |
| 22D5 | 283 | *Neo. tet./Neo. mod.* | *Neolamprologus modestus* |
| 22D5 | 284 | *Neo. tet./Neo. mod.* | *Neolamprologus modestus* |
| 22D5 | 285 | *Neo. tet./Neo. mod.* | *Neolamprologus modestus* |
| 22D5 | 286 | *Lepidiolamprologus attenuatus* | *Lepidiolamprologus attenuatus* |
| 22D5 | 287 | *Perissodus microlepis/Ple. str.* | *Plecodus straeleni* |
| 22D5 | 288 | *Neo. tet./Neo. mod.* | *Neolamprologus modestus* |
| 22D5 | 289 | *Lepidiolamprologus attenuatus* | *Lepidiolamprologus attenuatus* |
| 22D5 | 290 | *Neo. tet./Neo. mod.* | *Neolamprologus modestus* |
| 22D5 | 291 | *Interochromis loocki* | *Intloc/Petort* |
| 22D6 | 217 | *Tel. dho./Neo. sav.* | *Telmatochromis dhonti* |
| 22D6 | 218 | *Tel. dho./Neo. sav.* | *Telmatochromis sp.* |
| 22D6 | 219 |  |  |
| 22D6 | 220 | *Tel. dho./Neo. sav.* | *Telmatochromis sp.* |
| 22D6 | 221 | *Tel. dho./Neo. sav.* | *Telmatochromis sp.* |
| 22D6 | 222 | *Neo. caud./Lam. call./Lep. n'kambae* | *Altolamprologus sp.* |
| 22D6 | 223 | *Tel. dho./Neo. sav.* | *Telmatochromis temporalis* |
| 22D6 | 224 | *Lepidiolamprologus nkambae* | *Lamprologini* |
| 22D6 | 225 | *Tel. dho./Neo. sav.* | *Telmatochromis dhonti* |
| 22D6 | 226 | *Tel. dho./Neo. sav.* | *Telmatochromis temporalis* |
| 22D6 | 227 | *Tel. dho./Neo. sav.* | *Lamprologini* |
| 22D6 | 228 | *Tel. dho./Neo. sav.* | *Telmatochromis dhonti* |
| 22D6 | 229 | *Tel. dho./Neo. sav.* | *Telmatochromis dhonti* |
| 22D6 | 230 |  |  |
| 22D6 | 231 | *Tel. dho./Neo. sav.* | *Telmatochromis dhonti* |
| 22D6 | 232 | *Tel. dho./Neo. sav.* | *Telmatochromis sp.* |
| 22D6 | 233 | *Tel. dho./Neo. sav.* | *Telmatochromis dhonti* |
| 22D6 | 234 | *Oph. het./Cya. fur./Cun. lon.* | *Cyathopharynx furcifer* |
| 22D6 | 235 | *Petrochromis polyodon* | *Lamprologus callipterus* |
| 22D6 | 236 | *Alt. sp./Lam. cal.* | *Lamprologini* |
| 22D6 | 237 | *Tel. dho./Neo. sav.* | *Telmatochromis dhonti* |
| 22D6 | 238 | *Tel. dho./Neo. sav.* | *Telmatochromis sp.* |
| 22D6 | 239 | *Lepidiolamprologus nkambae* | *Telmatochromis sp.* |
| 22D6 | 240 | *Tel. dho./Neo. sav.* | *Telmatochromis sp.* |
| 22D6 | 241 |  |  |
| 22D6 | 242 |  |  |
| 22D6 | 243 | *Alt. sp./Lam. cal.* | *Lamprologus callipterus* |
| 22D6 | 244 |  |  |
| 22D6 | 245 |  |  |
| 22D6 | 246 |  |  |
| 22D6 | 247 |  |  |
| 22D6 | 248 |  |  |
| 22D6 | 292 | *Tel. dho./Neo. sav.* | *Telmatochromis sp.* |
| 22D6 | 293 | *Tel. dho./Neo. sav.* | *Telmatochromis sp.* |
| 22D6 | 294 | *Alt. sp./Lam. cal.* | *Lamprologini* |
| 22D6 | 295 | *Interochromis loocki* | *Tropheini* |
| 22D6 | 296 | *Petrochromis polyodon* | *Petrochromis polyodon* |
| 22D6 | 297 |  |  |
| 22D6 | 298 | *Petrochromis polyodon* | *Petrochromis polyodon* |
| 22D6 | 299 | *Tel. dho./Neo. sav.* | *Telmatochromis temporalis* |
| 21D5 | 97 | *Eretmodus cyanostictus* | *Eretmodus cyanostictus* |
| 21D5 | 98 | *Eretmodus cyanostictus* | *Eretmodus cyanostictus* |
| 21D5 | 99 | *Eretmodus cyanostictus* | *Eretmodus cyanostictus* |
| 21D5 | 100 | *Aulonocranus dewindti* | *Aulonocranus dewindti* |
| 21D5 | 101 | *Eretmodus cyanostictus* | *Eretmodus cyanostictus* |
| 21D5 | 102 | *Eretmodus cyanostictus* | *Eretmodus cyanostictus* |
| 21D5 | 103 | *Eretmodus cyanostictus* | *Eretmodus cyanostictus* |
| 21D5 | 104 | *Eretmodus cyanostictus* | *Eretmodus cyanostictus* |
| 21D5 | 105 | *Eretmodus cyanostictus* | *Eretmodus cyanostictus* |
| 21D5 | 106 | *Neo. lon./Cyp. lep. 'jumbo'* | *UNKNOWN CICHLID* |
| 21D5 | 107 |  |  |
| 21D5 | 108 | *Aulonocranus dewindti* | *Aulonocranus dewindti* |
| CYA9 | 626 | *Oreochromis tanganicae* | *UNKNOWN CICHLID* |
| CYA9 | 645 | *Lobochilotes labiatus* | *Lobochilotes labiatus* |
| CYA9 | 646 | *Lobochilotes labiatus* | *Tropheini??* |
| CYA9 | 647 | *Perissodus microlepis/Ple. str.* | *Perissodini?* |
| CYA9 | 792 | *Oreochromis tanganicae* | *Oreochromis tanganicae* |
| CYA9 | 793 | *Petrochromis polyodon* | *Petrochromis trewavasae* |
| CYA9 | 794 | *Oreochromis tanganicae* | *Oreochromis tanganicae* |
| CYA9 | 795 | *Tropheus sp. 'Nakaku'* | *Tropheus moori* |
| CYA9 | 796 | *Petrochromis polyodon* | *Petrochromis sp Texas* |
| CYA9 | 797 | *Ophthalmotilapia nasuta* | *Ophthalmotilapia nasuta* |
| CYA9 | 798 | *Tropheus sp. 'Nakaku'* | *Tropheus moori* |
| CYA9 | 799 | *Petrochromis polyodon* | *Lobochilotes labiatus* |
| CYA9 | 800 | *Petrochromis polyodon* | *Lobochilotes labiatus* |
| CYA9 | 801 | *Oreochromis tanganicae* | *Oreochromis tanganicae* |
| CYA9 | 802 | *Oreochromis tanganicae* | *Lobochilotes labiatus* |
| CYA9 | 803 | *Oreochromis tanganicae* | *Oreochromis tanganicae* |
| CYA9 | 804 | *Petrochromis polyodon* | *Lobochilotes labiatus* |
| CYA9 | 805 | *Petrochromis polyodon* | *Petrochromis trewavasae* |
| CYA9 | 806 | *Oreochromis tanganicae* | *Oreochromis tanganicae* |
| CYA9 | 807 | *Interochromis loocki* | *Astatotilapia burtoni* |
| CYA9 | 808 | *Oreochromis tanganicae* | *Oreochromis tanganicae* |
| CYA9 | 809 | *Oreochromis tanganicae* | *Oreochromis tanganicae* |
| CYA9 | 810 | *Petrochromis polyodon* | *Petrochromis trewavasae* |
| CYA9 | 811 | *Lobochilotes labiatus* | *Lobochilotes labiatus* |
| CYA9 | 812 | *Neolamprologus furcifer* | *Neolamprologus furcifer* |
| CYA9 | 813 | *Oreochromis tanganicae* | *Oreochromis tanganicae* |
| CYA9 | 814 | *Oreochromis tanganicae* | *Oreochromis tanganicae* |
| CYA9 | 815 | *Oreochromis tanganicae* | *Lobochilotes labiatus* |
| DAE7 | 768 | *Plecodus straeleni* | *Plestr/Permic* |
| DAE7 | 769 | *Simochromis diagramma* | *Lobochilotes labiatus* |
| DAE7 | 770 | *Lestradea stappersii* | *Lesper/Cunlon/Auldew* |
| DAE7 | 771 | *Lestradea stappersii* | *Ectodini* |
| DAE7 | 772 | *Lestradea stappersii* | *Lestradea stappersii* |
| DAH4 | 773 | *Tropheus sp. 'Nakaku'* | *Tropheus moori* |
| DAH4 | 774 | *Neolamprologus tretocephalus* | *Neolamprologus sexfasciatus* |
| DAH4 | 775 | *Petrochromis sp. 'macrognathus’* | *Petrochromis sp.* |
| DAH4 | 776 | *Tropheus sp. 'Nakaku'* | *Tropheus moori* |
| DAH4 | 777 | *Plecodus straeleni* | *Perissodus microlepis* |
| DAH4 | 778 |  |  |
| DAH4 | 779 | *Neolamprologus tretocephalus* | *Neolamprologus sexfasciatus* |
| DAH4 | 780 | *Perissodus microlepis* | *UNKNOWN CICHLID* |
| DAH4 | 781 | *Astatotilapia stappersii* | *Astatotilapia burtoni* |
| DAH4 | 782 | *Perissodus microlepis* | *Plecodus straeleni* |
| DAH4 | 783 | *Plecodus straeleni* | *Perissodus microlepis* |
| DAH4 | 784 | *Tropheus sp. 'Nakaku'* | *Tropheus moori* |
| DAH4 | 785 | *Tropheus sp. 'Nakaku'* | *Tropheus moori* |
| DAH4 | 786 | *Tropheus sp. 'Nakaku'* | *Tropheus polli* |
| DAH4 | 787 |  |  |
| DAH4 | 788 | *Neolamprologus tretocephalus* | *Neolamprologus sexfasciatus* |
| DAH4 | 789 |  |  |
| DAH4 | 790 | *Neolamprologus tretocephalus* | *Neolamprologus sexfasciatus* |
| DAH4 | 791 | *Tropheus sp. 'Nakaku'* | *Tropheus moori* |
| DBD7 | 629 | *Tropheus sp. 'Nakaku'* | *Tropheus moori* |
| DBD7 | 630 | *Tropheus sp. 'Nakaku'* | *Tropheus moori* |
| DBD7 | 631 | *Petrochromis polyodon* | *Tropheus duboisi* |
| DBD7 | 632 | *Simochromis diagramma* | *Lobochilotes labiatus* |
| DBD7 | 633 | *Tropheus sp./Lobochilotes labiatus* | *Lobochilotes labiatus* |
| DBD7 | 634 |  |  |
| DBD7 | 635 | *Tropheus sp. 'Nakaku'* | *Tropheus moori* |
| DBD7 | 636 | *Tropheus sp. 'Nakaku'* | *Tropheus sp.* |
| DBD7 | 637 |  |  |
| DBD7 | 638 | *Simochromis diagramma* | *Simochromis sp.* |
| DBD7 | 639 |  |  |
| DBD7 | 640 | *Petrochromis polyodon* | *Tropheus sp.* |
| DBD7 | 641 | *Petrochromis sp. 'macrognathus’* | *Simochromis diagramma* |
| DBD7 | 642 | *Petrochromis polyodon* | *Petrochromis sp. Texas* |
| DBD7 | 643 | *Tropheus sp. 'Nakaku'* | *Tropheus moori* |
| DBD7 | 644 |  |  |
| DDG5 | 696 |  |  |
| DDG5 | 697 |  |  |
| DDG5 | 698 |  |  |
| DDG5 | 699 |  |  |
| DDG5 | 700 |  |  |
| DDG5 | 701 |  |  |
| DDG5 | 702 |  |  |
| DDG5 | 703 |  |  |
| DDG5 | 704 |  |  |
| DDG5 | 705 | *Lobochilotes labiatus* | *Lobochilotes labiatus* |
| DDG5 | 706 | *Lobochilotes labiatus* | *Perissodini* |
| DDG5 | 707 | *Plecodus straeleni* | *Plecodus straeleni* |
| DDG7 | 744 | *Cyprichromis pavo* | *Neolamprologus similis* |
| DDG7 | 745 |  |  |
| DDG7 | 746 | *Perissodus microlepis* | *Plestr/Permic* |
| DDG7 | 747 | *Neolamprologus brichardi* | *Neolamprologus mondabu* |
| DDG7 | 748 | *Interochromis loocki* | *UNKNOWN CICHLID* |
| DDG7 | 749 | *Cyprichromis pavo* | *Cyprichromis pavo* |
| DDG7 | 750 | *Plecodus straeleni* | *Perissodus microlepis* |
| DDG7 | 751 | *Cyprichromis pavo* | *Neolamprologus obscurus* |
| DDG7 | 752 |  |  |
| DDG7 | 753 | *Xenochromis hecqui* | *Perissodini* |
| DDG7 | 754 | *Petrochromis fasciolatus* | *UNKNOWN CICHLID* |
| DDG7 | 755 | *Gnathochromis permaxillaris* | *UNKNOWN CICHLID* |
| DDG7 | 756 | *Xenotilapia bathyphila* | *Xenotilapia bathyphila* |
| DDG7 | 757 |  |  |
| DDG7 | 758 | *Plecodus straeleni* | *Perissodus microlepis* |
| DDG7 | 759 | *Cyprichromis pavo* | *Cyprichromis pavo* |
| DDG7 | 760 | *Perissodus microlepis* | *Plestr/Permic* |
| DDG7 | 761 | *Cyprichromis pavo* | *Cyprichromis pavo* |
| DDG7 | 762 |  |  |
| DDG7 | 763 | *Plecodus straeleni* | *Perissodus microlepis* |
| DDG7 | 764 | *Cyprichromis pavo* | *Cyprichromis pavo* |
| DDG7 | 765 |  |  |
| DDG7 | 766 |  |  |
| DDG7 | 767 | *Interochromis loocki* | *Interochromis loocki* |
| DFA1 | 648 | *Cyprichromis leptosoma* | *Cyprichromis leptosoma* |
| DFA1 | 649 | *Cyprichromis leptosoma* | *Cyprichromis leptosoma* |
| DFA1 | 650 | *Cyprichromis leptosoma* | *Cyprichromis leptosoma* |
| DFA1 | 651 |  |  |
| DFA1 | 652 | *Perissodus microlepis/Ple. str.* | *Plecodus straeleni* |
| DFA1 | 653 | *Cyprichromis leptosoma* | *Cyprichromis leptosoma* |
| DFA1 | 654 | *Cyprichromis leptosoma* | *Cyprichromis leptosoma* |
| DFA1 | 655 | *Cyprichromis leptosoma* | *Cyprichromis leptosoma* |
| DFA1 | 656 | *Plecodus straeleni* | *Plecodus straeleni* |
| DFA1 | 657 | *Cyprichromis leptosoma* | *Cyprichromis leptosoma* |
| DFA1 | 658 | *Cyprichromis leptosoma* | *Cyprichromis leptosoma* |
| DFA1 | 659 | *Cyprichromis leptosoma* | *Cyprichromis leptosoma* |
| DFA1 | 660 | *Cyprichromis leptosoma* | *Cyprichromis leptosoma* |
| DFA1 | 661 | *Cyprichromis leptosoma* | *Cyprichromis leptosoma* |
| DFA1 | 662 | *Cyprichromis leptosoma* | *Cyprichromis leptosoma* |
| DFA1 | 663 | *Cyprichromis leptosoma* | *Cyprichromis leptosoma* |
| DFA1 | 664 | *Plecodus straeleni* | *Plecodus straeleni* |
| DFA1 | 665 | *Cyprichromis leptosoma* | *Cyprichromis leptosoma* |
| DFA1 | 666 |  |  |
| DFA1 | 667 | *Cyprichromis leptosoma* | *Cyprichromis leptosoma* |
| DFA1 | 668 | *Boulengerochromis microlepis* | *UNKNOWN CICHLID* |
| DFA1 | 669 | *Cyprichromis leptosoma* | *Cyprichromis leptosoma* |
| DFA1 | 670 | *Perissodus microlepis/Ple. str.* | *Plestr/Permic* |
| DFA1 | 671 | *Cyprichromis leptosoma* | *Cyprichromis leptosoma* |
| DFG4 | 674 |  |  |
| DFG4 | 675 |  |  |
| DFG4 | 676 |  |  |
| DFG4 | 677 |  |  |
| DFG4 | 678 |  |  |
| DFG4 | 679 | *Cya. fur./Oph. het./Oph. ven.* | *Cyafur/Ophhet/Ophnas* |
| DFG4 | 680 |  |  |
| DFG4 | 681 | *Cya. fur./Oph. het./Oph. ven.* | *Cyafur/Ophhet/Ophnas* |
| DFG4 | 682 | *Cya. fur./Oph. het./Oph. ven.* | *Cyafur/Ophhet/Ophnas* |
| DFG4 | 683 | *Cya. fur./Oph. het./Oph. ven.* | *Cyafur/Ophhet/Ophnas* |
| DFG4 | 684 | *Cya. fur./Oph. het./Oph. ven.* | *Cyafur/Ophhet/Ophnas* |
| DFG4 | 685 | *Cya. fur./Oph. het./Oph. ven.* | *Cyafur/Ophhet/Ophnas* |
| DFG4 | 686 | *Cya. fur./Oph. het./Oph. ven.* | *Cyafur/Ophhet/Ophnas* |
| DFG4 | 687 | *Cya. fur./Oph. het./Oph. ven.* | *Cyafur/Ophhet/Ophnas* |
| DFG4 | 688 | *Cya. fur./Oph. het./Oph. ven.* | *Cyafur/Ophhet/Ophnas* |
| DFG4 | 689 | *Cya. fur./Oph. het./Oph. ven.* | *Cyafur/Ophhet/Ophnas* |
| DFG4 | 690 | *Cya. fur./Oph. het./Oph. ven.* | *Cyafur/Ophhet/Ophnas* |
| DFG4 | 691 | *Cya. fur./Oph. het./Oph. ven.* | *Cyafur/Ophhet/Ophnas* |
| DFG4 | 692 | *Cya. fur./Oph. het./Oph. ven.* | *Ophhet/Ophnas* |
| DFG4 | 693 |  |  |
| DFG4 | 694 |  |  |
| DFG4 | 695 | *Cya. fur./Oph. het./Oph. ven.* | *Ophhet/Ophnas* |
| DFG6 | 672 | *Perissodus microlepis/Ple. str.* | *Perissodus microlepis* |
| DFG6 | 673 | *Perissodus microlepis/Ple. str.* | *Perissodus microlepis* |
| DIF1 | 720 | *Plecodus straeleni* | *Plecodus straeleni* |
| DIF1 | 721 | *Plecodus straeleni* | *Plecodus straeleni* |
| DIF4 | 722 | *Petrochromis sp. 'macrognathus’* | *Petrochromis trewavasae* |
| DIF4 | 723 | *Petrochromis sp. 'macrognathus’* | *Petmac/Pet.sp.moshi* |
| DIF4 | 724 | *Petrochromis polyodon* | *Lobochilotes labiatus* |
| DIF4 | 725 | *Simochromis diagramma* | *Lobochilotes labiatus* |
| DIF4 | 726 | *Neolamprologus savoyri* | *Telmatochromis sp.* |
| DIF4 | 727 | *Simochromis diagramma* | *Loblab/Sim.sp* |
| DIF4 | 728 |  |  |
| DIF4 | 729 |  |  |
| DIF4 | 730 | *Eretmodus cyanostictus* | *Eretmodus cyanostictus* |
| DIF4 | 731 |  |  |
| DIF4 | 732 | *Petrochromis polyodon* | *Petrochromis polyodon* |
| DIF4 | 733 | *Eretmodus cyanostictus* | *Eretmodus cyanostictus* |
| DIF4 | 734 |  |  |
| DIF4 | 735 | *Petrochromis polyodon* | *Petrochromis polyodon* |
| DIF4 | 736 |  |  |
| DIF4 | 737 |  |  |
| DIF4 | 738 |  |  |
| DIF4 | 739 |  |  |
| DIF4 | 740 |  |  |
| DIF4 | 741 |  |  |
| DIF4 | 742 |  |  |
| DIF4 | 743 | *Simochromis diagramma* | *Tropheini* |
| DMH8 | 708 | *Neolamprologus pulcher* | *Neolamprologus mondabu* |
| DMH8 | 709 | *Cyprichromis pavo* | *Cyprichromis pavo* |
| DMH8 | 710 | *Cyprichromis pavo* | *Cyprichromis pavo* |
| DMH8 | 711 | *Neolamprologus brichardi* | *Neopul/Neobri* |
| DMH8 | 712 | *Neolamprologus pulcher* | *Neopul/Neobri* |
| DMH8 | 713 | *Neolamprologus pulcher* | *Neopul/Neobri* |
| DMH8 | 714 | *Cyprichromis pavo* | *Cyprichromis pavo* |
| DMH8 | 715 | *Neolamprologus pulcher* | *Neopul/Neobri* |
| DMH8 | 716 |  |  |
| DMH8 | 717 | *Neolamprologus pulcher* | *Neopul/Neobri* |
| DMH8 | 718 | *Neolamprologus christyi* | *Neolamprologus sp. eseki* |
| DMH8 | 719 | *Neolamprologus pulcher* | *Neopul/Neobri* |

**Table S6. GenBank Accession Numbers of Cichlid ND2 reference data set**

| **Species** | **GenBank Accession Nr.** |
| --- | --- |
| *Altolamprologus calvus* | EF462256 |
| *Altolamprologus compressiceps* | EF462257 |
| *Altolamprologus fasciatus* | EF191120 |
| *Altolamprologus sp. shell* | EF191107 |
| *Asprotilapia leptura* | AY337772 |
| *Astatotilapia burtoni* | AY930060 |
| *Aulonocranus dewindti* | AY337782 |
| *Baileychromis centropomoides* | AY682509 |
| *Bathybates fasciatus* | AY663732 |
| *Bathybates ferox* | AY663736 |
| *Bathybates graueri* | AY663726 |
| *Bathybates hornii* | AY663735 |
| *Bathybates leo* | AY663729 |
| *Bathybates minor* | AY663722 |
| *Bathybates vittatus* | AY663727 |
| *Benthochromis melanoide* | AY682512 |
| *Benthochromis tricoti* | AF317264 |
| *Boulengerochromis microlepis* | AF317229 |
| *Callochromis macrops* | AY337795 |
| *Callochromis pleurospilus* | AY337771 |
| *Callochromis stappersii* | AY337775 |
| *Cardiopharynx schoutedeni* | AY337791 |
| *Chalinochromis brichardi* | EF679241 |
| *Chalinochromis popeleni* | U07244 |
| *Ctenochromis benthicola* | JF900320 |
| *Ctenochromis horei* | EU753935 |
| *Cunningtonia longiventralis* | AY337780 |
| *Cyathopharynx furcifer* | AY337781 |
| *Cyphotilapia frontosa* | U07247 |
| *Cyprichromis leptosoma* | AF398224 |
| *Cyprichromis microlepidotus* | AY740346 |
| *Cyprichromis pavo* | AY740382 |
| *Cyprichromis zonatus* | AY740347 |
| *Ectodus descampsii* | AY337790 |
| *Enantiopus melanogenys* | AY337770 |
| *Eretmodus cyanostictus* | AF398220 |
| *Gnathochromis permaxillaris* | AY682522 |
| *Gnathochromis pfefferi* | U07248 |
| *Grammatotria lemairii* | AY337787 |
| *Greenwoodochromis bellcrossi* | AY682524 |
| *Greenwoodochromis christyi* | AY682528 |
| *Haplotaxodon microlepis* | EF437496 |
| *Haplotaxodon trifasciatus* | EF437492 |
| *Hemibates stenosoma* | AY663716 |
| *Interochromis loocki* | U07262 |
| *Julidochromis dickfeldi* | EF462230 |
| *Julidochromis marlieri* | AF398230 |
| *Julidochromis ornatus* | EF462229 |
| *Julidochromis regani* | EF462228 |
| *Julidochromis transcriptus* | EF462231 |
| *Lamprologus callipterus* | AF398226 |
| *Lamprologus kungweensis* | EF191084 |
| *Lamprologus laparogramma* | EF462278 |
| *Lamprologus lemairii* | EF462271 |
| *Lamprologus meleagris* | DQ055027 |
| *Lamprologus ocellatus* | EF462259 |
| *Lamprologus ornatipinnis* | EF462260 |
| *Lamprologus signatus* | EF191086 |
| *Lamprologus speciosus* | EF191102 |
| *Lamprologus teugelsi* | DQ055059 |
| *Lepidiolamprologus attenuatus* | EF462274 |
| *Lepidiolamprologus boulengeri* | DQ055040 |
| *Lepidiolamprologus elongatus* | EF462268 |
| *Lepidiolamprologus hecqui* | DQ055041 |
| *Lepidiolamprologus kendalli* | EF462269 |
| *Lepidiolamprologus nkambae* | EF462270 |
| *Lepidiolamprologus profundicola* | EF462276 |
| *Lestradea perspicax* | AY337765 |
| *Lestradea stappersii* | AY337792 |
| *Limnochromis abeelei* | AY682533 |
| *Limnochromis auritus* | AF398216 |
| *Limnochromis staneri* | AY682541 |
| *Limnotilapia dardenni* | EF679249 |
| *Lobochilotes labiatus* | U07254 |
| *Microdontochromis rotundiventralis* | AY337793 |
| *Microdontochromis tenuidentatus* | AY337784 |
| *Neolamprologus bifasciatus* | HM623809 |
| *Neolamprologus brevis* | EF462264 |
| *Neolamprologus brichardi* | AF398227 |
| *Neolamprologus buescheri* | EF462243 |
| *Neolamprologus calliurus* | DQ093112 |
| *Neolamprologus caudopunctatus* | EF462272 |
| *Neolamprologus christyi* | HM623826 |
| *Neolamprologus cunningtoni* | DQ055054 |
| *Neolamprologus cylindricus* | EF462224 |
| *Neolamprologus devosi* | EF437476 |
| *Neolamprologus falcicula* | EF462246 |
| *Neolamprologus furcifer* | EF679252 |
| *Neolamprologus gracilis* | HM623798 |
| *Neolamprologus helianthus* | DQ055013 |
| *Neolamprologus leleupi* | EF462251 |
| *Neolamprologus leloupi* | EF191103 |
| *Neolamprologus longicaudata* | EF462250 |
| *Neolamprologus longior* | HM623793 |
| *Neolamprologus marunguensis* | AY740390 |
| *Neolamprologus meeli* | DQ055051 |
| *Neolamprologus modestus* | DQ055012 |
| *Neolamprologus mondabu* | EF462242 |
| *Neolamprologus multifasciatus* | EF462266 |
| *Neolamprologus mustax* | EF462223 |
| *Neolamprologus niger* | AY740391 |
| *Neolamprologus nigriventris* | EF462239 |
| *Neolamprologus obscurus* | HM623824 |
| *Neolamprologus olivaceous* | AY740393 |
| *Neolamprologus pectoralis* | EF462238 |
| *Neolamprologus petricola* | HM623827 |
| *Neolamprologus prochilus* | EF462248 |
| *Neolamprologus pulcher* | EF462244 |
| *Neolamprologus savoryi* | EF462247 |
| *Neolamprologus sexfasciatus* | HM623828 |
| *Neolamprologus similis* | EF462261 |
| *Neolamprologus sp. eseki* | HM623794 |
| *Neolamprologus sp. kipili* | HM623802 |
| *Neolamprologus sp. ndobnoi* | HM623801 |
| *Neolamprologus splendens* | HM623799 |
| *Neolamprologus tetracanthus* | EF462220 |
| *Neolamprologus toae* | EF462222 |
| *Neolamprologus tretocephalus* | EF462219 |
| *Neolamprologus variostigma* | EF462253 |
| *Neolamprologus ventralis* | EF462233 |
| *Neolamprologus walteri* | HM623808 |
| *Neolamprologus wauthioni* | EF191118 |
| *Ophthalmotilapia boops* | AY337773 |
| *Ophthalmotilapia heterodonta* | EF679254 |
| *Ophthalmotilapia nasuta* | AY337783 |
| *Ophthalmotilapia ventralis* | AY337774 |
| *Oreochromis tanganicae* | AF317240 |
| *Paracyprichromis nigripinnis* | AY740339 |
| *Paracyprochromis brieni* | AY740378 |
| *Perissodus eccentricus* | EF437511 |
| *Perissodus microlepis* | AF398222 |
| *Perissodus paradoxus* | EF437500 |
| *Petrochromis ephippium* | JF900323 |
| *Petrochromis famula* | JF900324 |
| *Petrochromis fasciolatus* | JF900325 |
| *Petrochromis macrognathus* | AY930068 |
| *Petrochromis orthognathus* | U07262 |
| *Petrochromis polyodon* | JF900326 |
| *Petrochromis sp. katete* | GQ995748 |
| *Petrochromis sp. moshi* | GQ995765 |
| *Petrochromis sp. texas* | GQ995766 |
| *Petrochromis trewavasae* | GQ995761 |
| *Plecodus elaviae* | EF437504 |
| *Plecodus multidentatus* | EF437505 |
| *Plecodus straeleni* | EF437481 |
| *Pseudosimochromis curvifrons* | GQ995777 |
| *Reganochromis calliurus* | AY682544 |
| *Simochromis babaulti* | GQ995782 |
| *Simochromis diagramma* | AY930087 |
| *Simochromis marginatus* | AY930088 |
| *Simochromis pleurospilus* | GQ995783 |
| *Spathodus erythrodon* | DQ055008 |
| *Spathodus marlieri* | HM623786 |
| *Tanganicodus irsacae* | DQ055007 |
| *Telmatochromis bifrenatus* | AF398228 |
| *Telmatochromis brichardi* | EF462236 |
| *Telmatochromis dhonti* | EF679266 |
| *Telmatochromis temporalis* | EF462234 |
| *Telmatochromis vittatus* | EF462237 |
| *Telotrematocara macrostoma* | AY663715 |
| *Trematocara marginatum* | JF900327 |
| *Trematocara stigmaticum* | JF900328 |
| *Trematocara unimaculatum* | AF317268 |
| *Triglachromis otostigma* | AF398217 |
| *Tropheus brichardi* | AY930086 |
| *Tropheus duboisi* | AY930085 |
| *Tropheus moorii* | AY930093 |
| *Tropheus polli* | AY930084 |
| *Tylochromis polylepis* | U07268 |
| *Variabilichromis moorii* | DQ055016 |
| *Xenochromis hecqui* | EF437513 |
| *Xenotilapia bathyphila* | AY337789 |
| *Xenotilapia boulengeri* | HM135111 |
| *Xenotilapia caudafasciata* | AY337777 |
| *Xenotilapia flavipinnis* | AY337794 |
| *Xenotilapia longispinis* | AY337779 |
| *Xenotilapia ochrogenys* | AY337767 |
| *Xenotilapia sima* | AY337785 |
| *Xenotilapia sp. papiliosunflower* | AY337776 |
| *Xenotilapia spiloptera* | AY337788 |

**Figure S1. Reflectance Curves**

Mean spectral reflectance measurements from the different body regions (dark and light bars, dorsal and ventral positions) of the mimic *Plecodus straeleni* and the two models, *Neolamprologus sexfasciatus* (Model a) and *Cyphotilapia gibberosa* (Model b; see also Figure 1A). Note that, due to stress induced darkening, dorsal light bars appear similar in luminance (% reflectance) but not in chromaticity (shape of the curve) to the dark bars

**
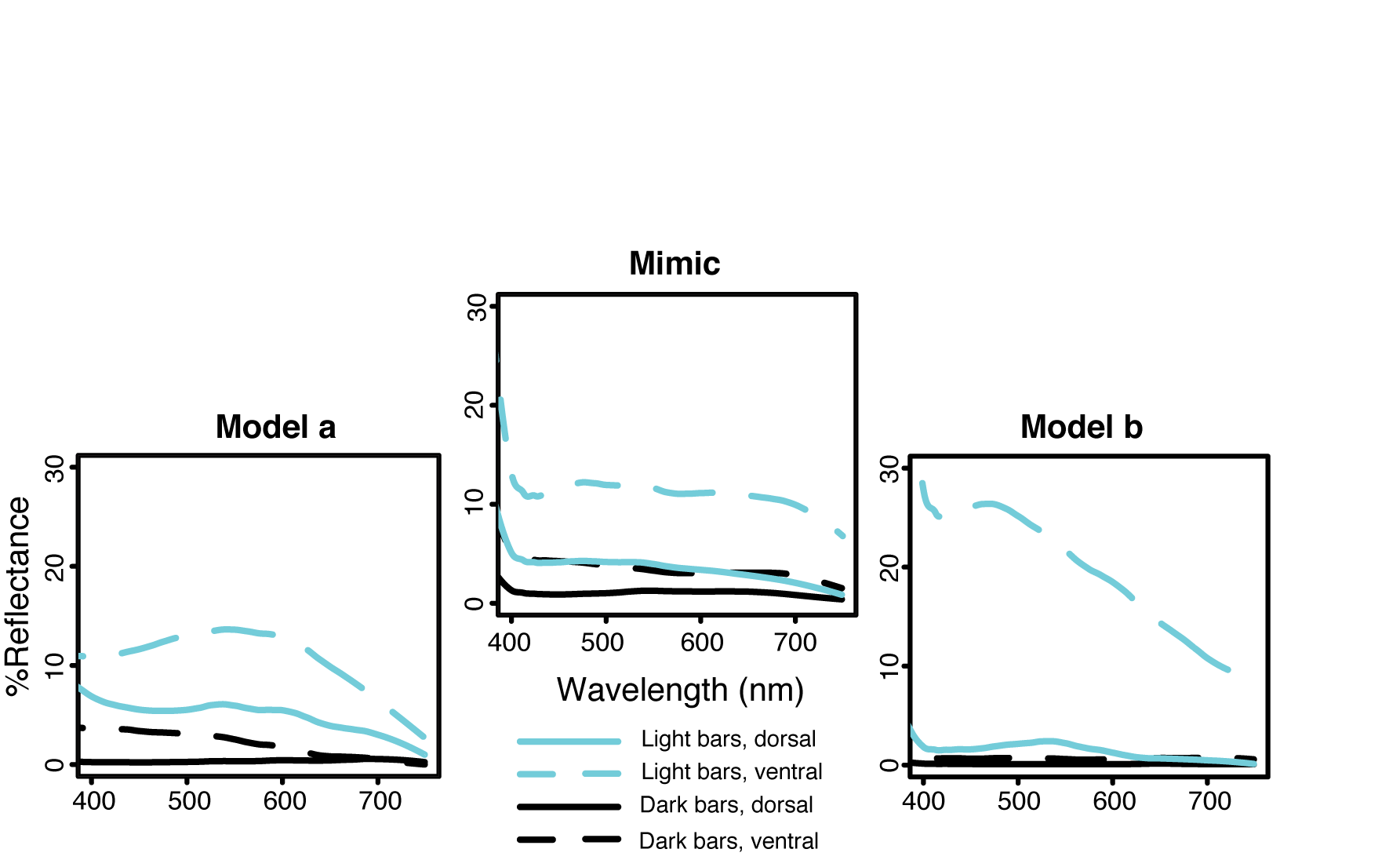
**

**Figure S2. Colour scoring of Tanganyikan cichlids by Detrended Correspondence Analysis**

Species involved in this mimicry complex exhibit a highly similar coloration that is distinct from all other Tanganyikan cichlids. The first two axes of a detrended correspondence analysis of binary colour scoring data are shown.

**Movie S1. The scale eater *Plecodus straeleni* and its models *Neolamprologus sexfasciatus* and *Cyphotilapia gibberosa***

(A) 0 min 04 sec – 0 min 15 sec: The scale eating cichlid *Plecodus straeleni* (the mimic) in its natural habitat

(B) 0 min 16 sec – 0 min 27 sec: *Neolamprologus sexfasciatus* (model A) in its natural habitat

(C) 0 min 28 sec – 0 min 39 sec: *Cyphotilapia gibberosa* (model B) in its natural habitat

(D) 0 min 40 sec – 1 min 04 sec: *P. straeleni* attacking other cichlid fish in Lake Tanganyika

(E) 1 min 05 sec – 1 min 21 sec: The mimic *P. straeleni* (to the left) and one of its models (*N. sexfasciatus*; to the right) interact with each other

(F) 1 min 22 sec – 1 min 32 sec: The mimic *P. straeleni* (left) passes the second model, *C. gibberosa* (to the right)

**Supplemental Experimental Procedures**

**Analysis of visual resemblance**

*Spectral reflectance measurements*

In order to compare the visual resemblance between the mimic and two of its co-occurring models in the South of Lake Tanganyika, we initially took spectral reflectance measurements of four different, distinct body regions of the fish (dark and light bars, each at dorsal and ventral areas). Adults of *P. straeleni* (males: N = 3; females: N = 5) and *C. gibberosa* (female: N = 1) were collected at Chituta bay, Southern Lake Tanganyika (8.72361 South, 31.15028 East), using scuba gear and gill nets. Additionally, we used *N. sexfasciatus* (males: N = 2) from our aquaria facilities at the University of Basel, Switzerland, which we originally caught from close by Chituta bay. Immediately after collection, reflectance spectra of the dorsal and ventral area of both dark and light bars were taken in the field/aquaria facilities using a JAZ Modular Portable spectrometer (Ocean Optics) with an integrated, pulsed Xenon lamp module (OCOJAZ-PX; wavelength range 300 – 980nm) and an OCOWS-1 diffuse reflection standard, following the methods described in [1]. Between three to six reflectance spectra were measured for each specimen and body area. Spectral files were inspected and processed using the R package Pavo [2]. Wavelengths were interpolated in 1 nm bins over a spectral range from 400 – 700 nm. Spectra from each body area and from individuals belonging to the same species were combined and averaged. Finally the data was smoothed and spectral curves were plotted for each species (Figure S1).

*Lake Tanganyika light environment*

The natural light environment under which the fish are perceived was measured at Isanga Bay, Southern Lake Tanganyika (8.65523 South, 31.19359 East) in September 2011 at a water depth of 7 m (H. Gante et al., unpublished). Illumination was measured using a USB2000 spectrometer attached to a PALM-SPEC computer running native software (Ocean Optics), enclosed in an underwater housing (Wills Camera Housings, Victoria, Australia). We used a shortened (60 cm) 1000 µm UV/visible optical fibre with a cosine corrector to provide an 180º hemisphere to measure both, down-welling (by pointing the fibre upwards) and side-welling light (by pointing the fibre horizontally towards or away from the shore). However, there was no substantial difference in our conclusion when using either of the illumination measurements and visual modelling results are consequently presented using down-welling light only (see below).

*Visual modelling of colour differences*

We used the Vorobyev-Osorio theoretical vision model [3] to estimate how similar, in terms of chromatic colour contrast (colour distance; ΔS) the mimic and its two models would appear under natural ambient light conditions (see above; Figure 1). ΔS was calculated following the methods in [4], assuming a 0.05 LWS noise threshold for the weber fraction (ω) and a cone receptor ratio of 1:2:2 (SWS:MWS:LWS), as commonly found for cichlids [5,6]. Most Lake Tanganyikan cichlids are known to express a set of three visual genes (opsins) corresponding to a middle- (green; SWS2B, RH2B, RH2Aα) or a long-wavelength (red; SWS2B, RH2Aα, LWS) palette [7]. The Tanganyikan opsins are functionally very conserved and highly similar to the ancestral riverine opsins of the Nile tilapia, *Oreochromis niloticus* [7]. Therefore, and in the absence of morphological measurements from Tanganyikan cichlids, we based the spectral sensitivities for the two palettes on the spectral sensitivities of *O. niloticus*: λ_max_ green-palette = 425, 472, 528 nm; λ_max_ red-palette = 425, 528, 561 nm [8].

*Colour scoring*

In order to evaluate and compare overall body coloration we used available data [9] and data new to this study. In total, 71 species were investigated, including *P. straeleni* and the two model species, the same set as in [10], of which 51 had previously been analysed for body coloration [9]. All major clades (i.e. tribes) of Tanganyikan cichlids were represented. We included pelagic, benthic and littoral species, sand-, mud- and rock-dwelling taxa, with diverse trophic ecologies. Our sampling method is unlikely to have biased the choice of species, since fishes were caught using angling and spear-fishing, as well as using gill- and hand-nets on SCUBA at a range of depths, at different times during day and night, at different times throughout the year, and over a 3 years period. Each species was scored for the presence or absence of 101 coloration traits using field photographs. Traits were defined according to typical hue, brightness, or patterning in separate regions of the adult fish body (e.g. “dorsal fin striped” or “operculum is yellow”; for detailed listings see Table S2). After removing invariant or unique traits from the data set (Table S3) we retained 90 traits, from which we calculated the principal axes of variation using detrended correspondence analysis [11]. To illustrate the relative resemblance in body coloration between mimic, models, and other Tanganyikan cichlid species we plotted the first two detrended correspondence axes (Figure S2).

**Transect survey**

In order to obtain information on the relative abundance of the mimic and its models, we performed a transect survey on SCUBA at seven sites at Lake Tanganyika in August 2014. At each site, the number of mimics (*P. straeleni*) and models (*C. gibberosa, N. sexfasciatus*) was recorded along a transect running from the shoreline to a water depth of 25 m. The transect was defined as the virtual line at a right angle to the shoreline measured with a SUUNTO diving compass, which was also used to exactly stay on the transect line. All sights of the three target species were recorded until a depth of 25 m was reached (due to safety issues, we did not continue further); all counts were performed by one diver (WS). *N. sexfasciatus* (model a) was the most abundant of the three species at all sites; *C. gibberosa* was only found at two sites (it typically occurs in greater depth) (see Table S4).

**Detailed molecular methods**

*Sampling*

Thirty-eight live specimens of *P. straeleni* were collected while SCUBA diving between 2008 and 2011 at 16 different locations in Lake Tanganyika, East Africa (see Table S1 for sampling dates and location). Fishes were sized, sexed, photographed and their digestive tract was immediately dissected and stored in ethanol. In the lab, we opened the digestive tracts and extracted the enclosed scales. Since ingested scales tend to clump together in a pile, they were first separated and rinsed in distilled water and carefully scrapped off to remove contaminating pieces of gut tissue. Scales were then rinsed again, quickly dried on ﬁlter paper and transferred to an individual vial. As the digestive state of the extracted scales did not permit a straightforward morphological identification, we used a PCR-based approach to identify prey species.

*DNA extraction from individual scales*

To the individual scales, we added 155 µL of lysis buffer (1M Tris pH 8.0, 0.5M EDTA pH 8.0, 5M NaCl and SDS 2% modiﬁed from [12]), and 5 µL of Proteinase K (10 mg/ml; Roche Diagnostics). Lysis was performed in a thermal shaker at 42°C, 550 rpm for 6 hours. We then applied a phenol-chloroform-isoamyl alcohol (25:24:1) extraction, followed by two subsequent chloroform-isoamyl alcohol (24:1) washing steps. Alternatively (with comparable results), we used a salt extraction method (by addition of 90 µL of 5M NaCl) to salt out proteins. DNA was precipitated with ammonium acetate 2.5M, 3 volumes of pure ethanol and 1 µL of glycogen 20 mg/ml (Roche Diagnostics). For very small scales, precipitation was done overnight at room temperature to ensure maximum recovery. Sample tubes were then spun down for 30 min to 1 hour at 13’000 g; the resulting DNA pellets were washed two times with 70% ethanol. After air-drying for 15 min, the pellets were resuspended in 20 to 50 µL TE buffer (100 mM Tris-Cl pH 8.0, 10 mM EDTA pH 8.0). DNA yield and quality was checked with a spectrophotometer (Nanodrop 2000, Thermo Scientiﬁc).

*PCR amplification*

As a species-specific marker locus, we chose the Subunit 2 of the mitochondrial NADH-Dehydrogenase (ND2) gene. The ND2 gene is particularly suited for resolving species-level relationships between cichlids from Lake Tanganyika, and it is also the locus for which the most complete reference dataset is available, covering more than 90% of all cichlid species from this lake [10,13,14]. Using a mitochondrial DNA marker has the additional advantage that it exists in multiple copies per cell, thereby increasing detection probability in the cases where the DNA was degraded due to the conditions in the digestive tracks of *P. straeleni*. Indeed, visual inspection of DNA extracts from scales on a 2% agarose gel revealed degradation into small fragments in many samples. We thus decided to design primers that would amplify smaller fragments of ND2 (407 bp and 304 bp, respectively), instead of aiming for the entire gene sequence (1047 bp). The primers used to amplify scale DNA were ND2-327 (5’-CCC TCT TCA TGC TTG ACT CC-3’) and ND2-733 (5’-GGG GTG TGA GAG CTG TTA GG-3’) for the 407 bp fragment, or, alternatively, ND2-409 (5’-GCC CCC TTC GCC CTA ATT CT-3’) and ND2-712 (5’-CGG GGG CTT TTG TTC AGG AT-3’) for the 304 bp fragment. In addition, to inhibit PCR amplification of scale-eater DNA, we designed blocking oligonucleotides [15] that specifically anneal to *P. straeleni* DNA but not to other cichlids: Perplex 488 blocH (5’-ctg GCC CTT GTT GGG GGC TGA ttt-3’) and Perplex 617 blocL (5’-aaa CAT AAT GAA GTA GGT AAG AAG GGT ctc-3’). They possess three overhanging non-complementary nucleotides at both ends that impede the progression of DNA polymerase along the DNA strand (when using a polymerase that lacks 3’ to 5’ exonuclease activity). PCR reactions were performed in a total volume of 12.5 µL, with both forward and reverse primers at a final concentration of 0.12 µM, 2 µM MgCl_2_, 0.25 µM of each of the four dNTPs, 0.8 µM of blocking oligonucleotides, and 0.25 units of AmpliTaq (Applied Biosystems). The conditions for PCR amplification were 95°C (1 min), then 40 cycles at 95°C (15 s) / 55°C (for ND2-327/733) or 58°C (for ND2-409/712) (15 s) / 72°C (15 s), and a final extension at 72°C (6 min). For one class of morphologically distinct scales found in two of the stomachs, which did not amplify with our cichlid specific ND2 primers, we switched to another mitochondrial marker, the cytochrome c oxidase, subunit 1 (COI), using published primers for discriminating fish species [16]. In this case, the 20 µL PCR reaction mix contained 10 µL of Phusion Master Mix with High Fidelity Buffer (New England Biolabs), 0.15 µL of the primers FishF2 and FishR2 and 2.5 µL of template DNA. Cycling conditions were 98°C (30 s), then 35 cycles at 98°C (10 s), 54°C (30 s), 72°C (25 s) and a final extension at 72°C (8 min). Amplification of *P. straeleni* COI region failed with this primer pair, circumventing the problem of contamination by gut tissue. All PCR products were checked with agarose gel electrophoresis.

*Sanger sequencing*

Prior to sequencing, 1 µL of PCR product was enzymatically cleaned with ExoSAP-IT (Affymetrix). For cycle sequencing we added 1 µL of BigDye 3.1 Ready reaction mix (Applied Biosystems) and 0.5 µL of primer. To increase sequence recovery success, we sequenced each PCR product in both directions. Cycling conditions were: 94°C (1 min), then 40 cycles at 94°C (1 min), 60°C (1 min 30 s). We then purified the entire product using the BigDye XTerminator purification kit (Applied Biosystems) and sequenced it on an AB3130*xl* Genetic Analyser (Applied Biosystems). Finally, the resultant sequences were inspected by eye and aligned to a reference ND2 dataset using CodonCode Aligner v3.7 (CodonCode Corporation). In total, we obtained 482 ND2 sequences out of the 815 extracted scales (59.1 %). All the 128 sequences obtained with COI primers were highly similar and turned out, according to a BLAST search, to belong to *Mastacembelus ellipsifer*, a spiny eel endemic to Lake Tanganyika. Table S2 provides a detailed list of our results.

*Phylogenetic analyses*

To assign unknown scale sequences to a particular cichlid species, we opted for a phylogenetic approach in addition to BLAST searches, as – due to the close relatedness of many of the cichlid species in Lake Tanganyika – an unambiguous assignment to reference sequences with BLAST was not possible. First, we collapsed our ND2 data set into haplotypes using the packages Ape 2.7-1  [17] and Pegas 0.3-4 [18] in R [19], resulting in a total of 327 different haplotypes. We then performed a maximum parsimony analysis in MEGA 5.0 [20] using a dataset including all 180 references sequences and the partial ND2 sequences from scale DNA after collapsing. Note that due to the non-overlap of some of the shorter scale DNA sequences, we could only use parsimony for this analysis. The analysis revealed that the scale-derived sequences grouped into eight distinct clades. We thus compiled eight subsets with query and reference sequences, and analysed them in more details using PAUP* [21]. Depending on the resolution of the phylogenetic analyses, each unknown scale sequence was assigned to a particular cichlid species (61% of all sequences), a genus (13%) or a tribe (9%). Especially the shorter sequences could only be assigned into genera or tribes; 4% of all sequences could not be assigned and were treated as “unknown”. All sequences assigned to either *P. straeleni*, *Perissodus microlepis* or to the Perissodini tribe (13%) were treated as exogenous *P. straeleni* DNA and excluded from further analyses.

**Diet Analyses**

*Dietary Indexes Calculation*

To characterize the distribution and abundance of prey species, we calculated two separate dietary indexes [22]: The percent frequency of occurrence of prey (*%FO*) indicates how frequently a prey is encountered in a stomach, while the average percent number (*%N*) describes the average representation of a given prey within one stomach, in our case the number of scales. We used the following formulas:

Percent Frequency of Occurrence of prey *i*: ${FO}_{i}\%= n_{i}/n$

$n_{i}$ number of stomachs containing prey *i*

$n$ total number of stomachs studied

Average Percent Number of prey *i*: $\%N_{i}=\left( \sum_{j=1}^{n} \%N_{ij} \right)/n$

$\%N_{ij}$ number of scales belonging to prey *i* divided by the total nr. of scales found in stomach *j*

$n$ total number of stomachs studied

*Individual Specialization*

We ran the package RInSp 2.15.3 in R [19,23] to characterize diet overlap or specialization between *P. straeleni* individuals. More specifically, we wanted to know whether some stomachs contained disproportionate numbers of scales of the same species. Using the data matrix containing prey species counts in columns (either categorized as tribes or left as species) and stomach individuals in rows, we computed the average pairwise overlap similarity. However, due to limited sampling size, estimation of likelihood measures of niche breadth and overlap were small and associated probabilities were not robust (data not shown).

**Supplemental References**

1. Gray, S. M., Hart, F. L., Tremblay, M. E. M., Lisney, T. J. & Hawryshyn, C. W. 2011 The effects of handling time, ambient light, and anaesthetic method, on the standardized measurement of fish colouration. *Can. J. Fish. Aquat. Sci.* **68**, 330–342. (doi:10.1139/F10-151)

2. Maia, R., Eliason, C. M., Bitton, P.-P., Doucet, S. M. & Shawkey, M. D. 2013 pavo : an R package for the analysis, visualization and organization of spectral data. *Methods Ecol. Evol.* **4**, 906–913. (doi:10.1111/2041-210X.12069)

3. Vorobyev, M. & Osorio, D. 1998 Receptor noise as a determinant of colour thresholds. *Proc. R. Soc. B* **265**, 351–358. (doi:10.1098/rspb.1998.0302)

4. Cortesi, F. & Cheney, K. L. 2010 Conspicuousness is correlated with toxicity in marine opisthobranchs. *J. Evol. Biol.* **23**, 1509–1518. (doi:10.1111/j.1420-9101.2010.02018.x)

5. Fernald, R. D. 1981 Chromatic organization of a cichlid fish retina. *Vision Res.* **21**, 1749–1753. (doi:10.1016/0042-6989(81)90207-8)

6. Dalton, B. E., Loew, E. R., Cronin, T. W. & Carleton, K. L. 2014 Spectral tuning by opsin coexpression in retinal regions that view different parts of the visual field. *Proc. R. Soc. B* **281**, 20141980. (doi:10.1098/rspb.2014.1980)

7. O’Quin, K. E., Hofmann, C. M., Hofmann, H. A. & Carleton, K. L. 2010 Parallel Evolution of opsin gene expression in African cichlid fishes. *Mol. Biol. Evol.* **27**, 2839–2854. (doi:10.1093/molbev/msq171)

8. Spady, T. C., Parry, J. W. L., Robinson, P. R., Hunt, D. M., Bowmaker, J. K. & Carleton, K. L. 2006 Evolution of the cichlid visual palette through ontogenetic subfunctionalization of the opsin gene arrays. *Mol. Biol. Evol.* **23**, 1538–1547. (doi:10.1093/molbev/msl014)

9. Muschick, M., Nosil, P., Roesti, M., Dittmann, M. T., Harmon, L. & Salzburger, W. 2014 Testing the stages model in the adaptive radiation of cichlid fishes in East African Lake Tanganyika. *Proc. R. Soc. B* **281**, 20140605. (doi:10.1098/rspb.2014.0605)

10. Muschick, M., Indermaur, A. & Salzburger, W. 2012 Convergent evolution within an adaptive radiation of cichlid fishes. *Curr. Biol.* **22**, 1–7. (doi:10.1016/j.cub.2012.10.048)

11. Hill, M. O. & Gauch, H. G. 1980 Detrended correspondence analysis: An improved ordination technique. *Vegetatio* **42**, 47–58. (doi:10.1007/BF00048870)

12. Miller, S. A., Dykes, D. D. & Polesky, H. F. 1988 A simple salting out procedure for extracting DNA from human nucleated cells. *Nucleic Acids Res.* **16**, 1215. (doi:10.1093/nar/16.3.1215)

13. Salzburger, W., Meyer, A., Baric, S., Verheyen, E. & Sturmbauer, C. 2002 Phylogeny of the Lake Tanganyika cichlid species flock and its relationship to the Central and East African haplochromine cichlid fish faunas. *Syst. Biol.* **51**, 113–35. (doi:10.1080/106351502753475907)

14. Day, J. J., Cotton, J. A. & Barraclough, T. G. 2008 Tempo and mode of diversification of lake Tanganyika cichlid fishes. *PLoS One* **3**, e1730. (doi:10.1371/journal.pone.0001730)

15. Vestheim, H. & Jarman, S. N. 2008 Blocking primers to enhance PCR amplification of rare sequences in mixed samples - a case study on prey DNA in Antarctic krill stomachs. *Front. Zool.* **5**, 12. (doi:10.1186/1742-9994-5-12)

16. Brown, K. J., Rüber, L., Bills, R. & Day, J. J. 2010 Mastacembelid eels support Lake Tanganyika as an evolutionary hotspot of diversification. *BMC Evol. Biol.* **10**, 188. (doi:10.1186/1471-2148-10-188)

17. Paradis, E., Claude, J. & Strimmer, K. 2004 APE: Analyses of Phylogenetics and Evolution in R language. *Bioinformatics* **20**, 289–290. (doi:10.1093/bioinformatics/btg412)

18. Paradis, E. 2010 pegas: an R package for population genetics with an integrated-modular approach. *Bioinformatics* **26**, 419–20. (doi:10.1093/bioinformatics/btp696)

19. R Development Core Team 2011 *R: A language and environment for statistical computing*. Vienna, Austria: R Foundation for Statistical Computing.

20. Tamura, K., Peterson, D., Peterson, N., Stecher, G., Nei, M. & Kumar, S. 2011 MEGA5: molecular evolutionary genetics analysis using maximum likelihood, evolutionary distance, and maximum parsimony methods. *Mol. Biol. Evol.* **28**, 2731–2739. (doi:10.1093/molbev/msr121)

21. Swofford, D. L. 2003 *PAUP*. Phylogenetic Analysis Using Parsimony (*and Other Methods)*. Version 4. Sunderland, Massachusetts: Sinauer Associates.

22. Brown, S. C., Bizzarro, J. J., Cailliet, G. M. & Ebert, D. A. 2011 Breaking with tradition: redefining measures for diet description with a case study of the Aleutian skate *Bathyraja aleutica* (Gilbert 1896). *Environ. Biol. Fish.* **95**, 3–20. (doi:10.1007/s10641-011-9959-z)

23. Zaccarelli, N., Bolnick, D. I. & Mancinelli, G. 2013 RInSp: An R package for the analysis of individual specialization in resource use. *Methods Ecol. Evol.* **4**, 1018–1023. (doi:10.1111/2041-210X.12079)
