# Supplementary material for: A complex mode of aggressive mimicry in a scale-eating cichlid fish
Source: Biol Lett. 2015 Sep;11(9):20150521. doi: 10.1098/rsbl.2015.0521 (PMC4614428; doi:10.1098/rsbl.2015.0521)
Supplement: Boileau_et_al_media.docx [file rsbl20150521supp2.docx]

Feeding on the scales of other species belongs to the most curious foraging strategies in fish. The exceptionally diverse species-flock of cichlid fishes of East African Lake Tanganyika contains six scale-eating species. They closely resemble other cichlid species, and it has been suggested that they imitate their models to feed upon their scales. We determined the DNA sequence of hundreds of scales extracted from the intestinal tracts of the scale-eater *Plecodus straeleni* and show that – contrary to the prevailing assumption – it feeds on a variety of prey species, adopting the strategy of a ‘wolves in sheep’s clothing eating goats’.
